# Cyclic jetting enables microbubble-mediated drug delivery

**DOI:** 10.1038/s41567-025-02785-0

**Published:** 2025-02-21

**Authors:** Marco Cattaneo, Giulia Guerriero, Gazendra Shakya, Lisa A. Krattiger, Lorenza G. Paganella, Maria L. Narciso, Outi Supponen

**Affiliations:** 1 Institute of Fluid Dynamics, ETH Zürich, Zürich, Switzerland; 2 Department of Obstetrics, University Hospital Zürich, University of Zürich, Zürich, Switzerland; 3 Institute of Energy and Process Engineering, ETH Zürich, Zürich, Switzerland; 4 Institute for Mechanical Systems, ETH Zürich, Zürich, Switzerland; 5 Swiss Federal Laboratories for Materials Science and Technology (EMPA), Dübendorf, Switzerland

**Keywords:** Fluid dynamics, Biophysics

## Abstract

The pursuit of targeted therapies capable of overcoming biological barriers, including the blood–brain barrier, has spurred the investigation of stimuli-responsive microagents that can improve therapeutic efficacy and reduce undesirable side effects. Intravenously administered, ultrasound-responsive microbubbles are promising agents with demonstrated potential in clinical trials, but the mechanism underlying drug absorption remains unclear. Here we show that ultrasound-driven single microbubbles puncture the cell membrane and induce drug uptake through stable cyclic microjets. Our theoretical models successfully reproduce the observed bubble and cell dynamic responses. We find that cyclic jets arise from shape instabilities, as opposed to classical inertial jets that are driven by pressure gradients, enabling microjet formation at mild ultrasound pressures below 100 kPa. We also establish a threshold for bubble radial expansion beyond which microjets form and facilitate cellular permeation and show that the stress generated by microjetting outperforms previously suggested mechanisms by at least an order of magnitude. Overall, this work elucidates the physics behind microbubble-mediated targeted drug delivery and provides the criteria for its effective and safe application.

## Abstract (web summary)

Ultrasound-driven microbubbles are promising candidates for drug delivery, but the mechanism of action is unclear. Now, single microbubbles induce drug uptake through cyclic microjets formed at mild ultrasound pressures via interfacial instability.

## Main

Targeted drug delivery holds the potential to revolutionize healthcare by enhancing the precision of drug administration and, thus, minimizing side effects^1^. By using specially engineered vascular carriers, drugs are encapsulated, transported and released at designated sites within the body^2,3^. Despite the ability of this approach to increase drug accumulation at targeted regions, its therapeutic efficacy is hindered by biological barriers, particularly the endothelium and the blood–brain barrier (BBB). They tightly regulate the molecular passage between the bloodstream and tissues, thereby limiting the drug bioavailability^4^.

**Fig. 1:**
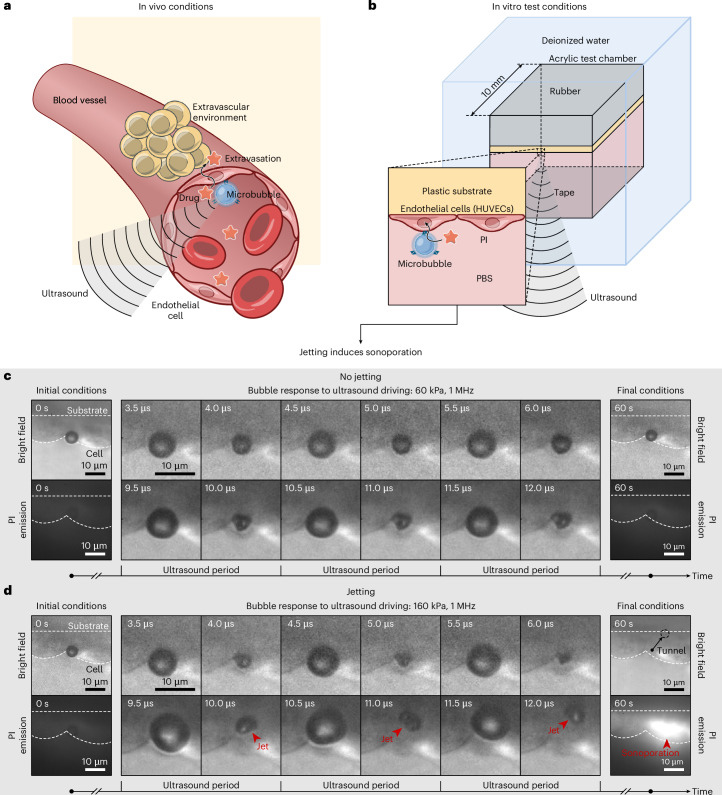
Targeted drug delivery mediated by ultrasound-responsive microbubbles. **a**, Schematic illustrating in vivo extravascular drug delivery induced by the mechanical action exerted by an ultrasound-driven microbubble. **b**, Schematic depicting the in vitro test model used to study the bubble–cell dynamics and the corresponding intracellular drug uptake with a side-view perspective. Methods and Extended Data Fig. 1 provide details about the experimental setup. **c**,**d**, Response of a microbubble (equilibrium radius *R*_0_ = 3 μm) to varying ultrasound pressure amplitudes and the corresponding model drug uptake. In **c**, the applied ultrasound pressure induces spherical oscillations in the bubble, followed by asymmetric deformation (*p*_a_ = 60 kPa, *f* = 1 MHz). However, this does not result in cell membrane poration and drug uptake. In **d**, a higher ultrasound pressure causes the bubble to develop cyclic piercing microjets directed towards the cell (*p*_a_ = 160 kPa, *f* = 1 MHz), resulting in cell membrane poration and drug uptake.

Ultrasound-responsive agents, such as phospholipid-coated microbubbles, offer solutions for enhancing specificity and overcoming biological barriers in drug delivery^5–9^. These agents, either co-administered with drugs in the systemic circulation or directly conjugated to them, are actuated with spatial precision by focused ultrasound systems^10^. Ultrasound induces cyclic oscillations in the bubbles, generating mechanical stresses that temporarily open biological barriers, enabling drug delivery across them^11^ (Fig. 1a).

As of today, ultrasound-activated microbubbles are the only non-invasive, localized and reversible method for opening the BBB and delivering drugs to the brain^12^. This technique holds promise for treating neurodegenerative disorders such as Alzheimer’s and Parkinson’s diseases^13–15^, brain tumours^16–21^ and amyotrophic lateral sclerosis in humans^22^. Additionally, it shows potential for treating solid tumours^23,24^, myocardial infarction^25^ and atherosclerosis^26^.

Despite promising clinical results, the physical mechanism by which microbubbles enhance biological barrier permeability remains unclear. Proposed mechanisms include acoustic streaming^27^, inertial jetting^28^, normal impact pressure^29^ and viscous shear stress^30^. The lack of consensus underscores the formidable challenge of directly observing bubble behaviour and correlating it with drug uptake, a crucial step for ensuring the safety of microbubble-mediated drug delivery.

Cell monolayers on rigid substrates are the primary in vitro platform for monitoring membrane integrity and drug uptake with ultrasound-driven microbubbles^29–33^. Ex vivo tissues^34,35^ and in vivo embryos^36^ have also been used to investigate the dynamics of microbubbles within vascular structures, but the resulting drug uptake has not been analysed. Current investigations using cell monolayers are constrained to a top-down view, which provides an incomplete picture of the bubble–cell interplay. In this study, we adopt a side-view perspective to explore the underlying physics. This approach presents substantial challenges due to restricted optical access, which we address through a carefully designed experimental setup and test samples. Our viewpoint on the problem enables us to uncover key insights into the physics of microbubble-mediated drug delivery. We anticipate our investigation to guide the future developments of this technology.

## Bubble jetting and sonoporation

**Extended Data Fig. 1:**
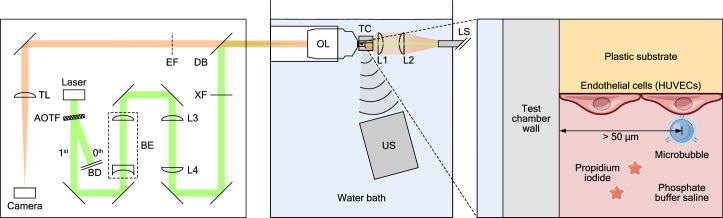
Schematic of the experimental setup. (AOTF) Acousto-optic tunable filter, (BD) Beam dump, (BE) Beam expander, (DB) Dichroic beamsplitter, (EF) Emission filter, (L1-L4) Lens, (LS) Light source, (OL) Objective lens, (TC) Test chamber, (TL) Tube lens, (US) Ultrasound transducer, (XF) Excitation filter.

To enable the side-view visualizations of drug delivery facilitated by individual microbubbles, we culture human umbilical vein endothelial cells (HUVECs) on a plastic substrate that we position within a custom-designed test chamber. The chamber features an acoustically transparent base and optically transparent sides. We fill the chamber with a solution of phosphate-buffered saline (PBS), propidium iodide (PI) and phospholipid-coated microbubbles (1–4 μm in radius). Microbubbles adhere to cells through flotation. The chamber is immersed in a water bath (Fig. 1b). We use a single ultrasound pulse (frequency *f* = 1 MHz; 20 cycles) with a ramp profile, directed from the bottom, to drive the microbubbles. We capture the bubble response under varying ultrasound pressures using a custom-built side-view ×200 microscope recording at 10 million frames per second and assess the cell membrane permeabilization by observing the intracellular fluorescence of PI, which serves as a model drug (Methods and Extended Data Fig. 1).

At a mild ultrasound pressure (*p*_a_ = 60 kPa), a single microbubble (equilibrium radius *R*_0_ = 3 µm) in contact with a cell undergoes alternating phases of expansion and compression and maintains its spherical shape (Fig. 1c and Supplementary Video 1; 3.5–6.0 μs). Over time, the microbubble starts to exhibit non-spherical compression phases, forming microjets (Fig. 1c and Supplementary Video 1; 9.5–12.0 μs). These jets are aimed at the cell but lack sufficient momentum to pierce the bubble and impact the cell on the opposite side. The cell membrane remains undamaged, as indicated by the absence of intracellular fluorescence (Fig. 1c; 60 s). At a higher ultrasound pressure (*p*_a_ = 160 kPa), the same microbubble experiences a larger radial excursion and now develops piercing cyclic jets that hammer the cell at each compression phase (Fig. 1d and Supplementary Video 2; 9.5–12.0 μs). This response results in cell membrane poration and PI uptake, evidenced by the intense fluorescent emission (Fig. 1d; 60 s). The process of mechanically opening the cell membrane using microbubbles and ultrasound is known as sonoporation^37^.

**Extended Data Fig. 2:**
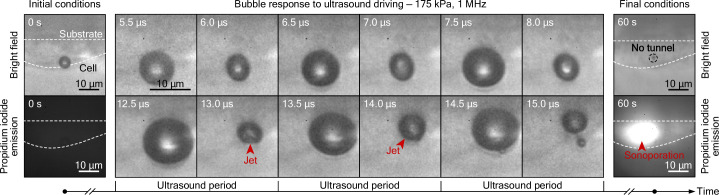
Cyclic microjetting of a microbubble resulting in no transendothelial tunnel formation. Cyclic microjets generated by a microbubble (*R*_0_ = 3.0 *μ*m) induce cell membrane poration, facilitating drug uptake (*p*_a_ = 175 kPa, *f* = 1 MHz). Yet, in this instance, the bubble motion does not form a transendothelial tunnel, as the cell deformation recovers and the bubble returns to its initial position when the ultrasound pulse stops.

Previous studies have shown that sonoporation can cause the opening of cell–cell contacts^38^ and, in some cases, lead to the formation of transendothelial tunnels—transcellular perforations that pierce both the apical and basal cell membranes^39,40^—thereby facilitating transcellular drug delivery into the extravascular space. Although the side-view perspective does not allow the visualization of cell–cell openings, it does reveal that in the current case of successful sonoporation, bubble activity not only deforms and punctures the apical cell membrane but also induces the formation of a transendothelial tunnel. This is evident from the microbubble position after the ultrasound pulse, as it rests deflated against the plastic substrate at the basal membrane level (Fig. 1d; 60 s). However, tunnel formation does not occur in all sonoporation events. If the cell membrane damage is insufficient to internalize the bubble, it returns to its initial position when the ultrasound pulse ends (Extended Data Fig. 2 and Supplementary Video 3). Nevertheless, whether through tunnelling or reversible cell deformation, the bubble has the potential to reach and perforate the basal cell membrane, thereby enabling drug transport into the extravascular space.

When ultrasound is applied without the presence of microbubbles, no sonoporation events occur at the tested ultrasound pressures (up to 1 MPa), even after tens of repeated pulses.

## Characterization of bubble dynamics

**Fig. 2:**
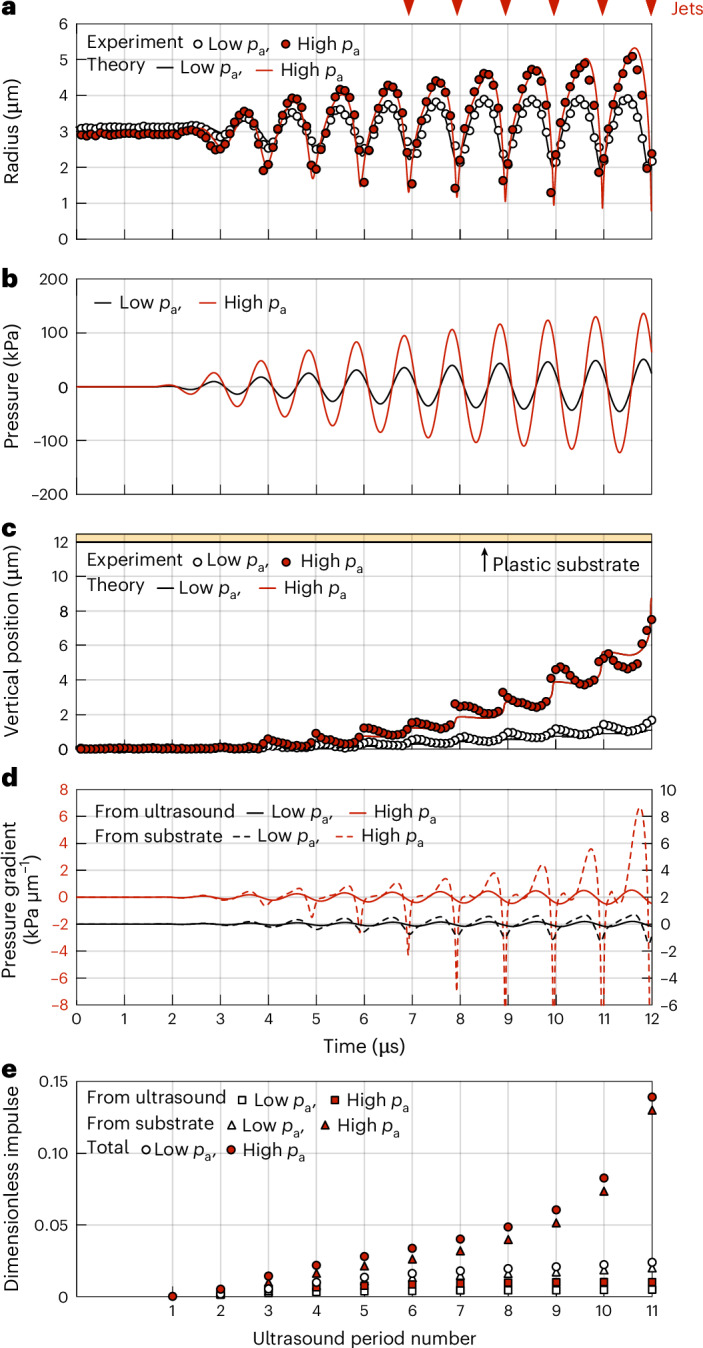
Characterization of bubble dynamics. In the legend, low *p*_a_ and high *p*_a_ refer to Fig. 1c,d, respectively. The red arrows at the top indicate when jets occur in the high-*p*_a_ case. Methods provides details about the theoretical models. **a**, Experimental and theoretical radial motion of the microbubble. The uncertainty in the bubble radius measurement corresponds to half the pixel size (80 nm). **b**, Ultrasound pulse driving the microbubble. The pulse shape is recorded with a hydrophone. The pulse amplitude is inferred as the only fitting parameter from the corresponding radius–time curve in **a**. **c**, Experimental and theoretical vertical positions of the microbubble centroid. The yellow area represents the plastic substrate. The uncertainty in the bubble position measurement corresponds to half the pixel size (80 nm). **d**, Time evolution of the pressure-gradient contributions from the ultrasound pulse and plastic substrate at the bubble position. The curves for the two ultrasound pressure cases are vertically offset to enhance visibility. **e**, Evolution of the dimensionless impulse contributions from the ultrasound, the plastic substrate and the sum thereof over ultrasound cycles. Source data

The time evolution of the microbubble radius in the cases depicted in Fig. 1c,d is extracted from video recordings and compared with theoretical predictions (Fig. 2a). The theoretical model incorporates a modified Rayleigh–Plesset equation for the liquid phase^41^, the Zhou thermal model for the gaseous phase^42^ and the Marmottant model for the phospholipid coating^43^ (Methods). Despite assuming spherical symmetry, the model agrees well with observations, as the bubble remains mostly spherical due to the mild constriction of the soft cellular monolayer, deviating only during the final instants of the compression phase to form jets (Fig. 2b). The ultrasound pressure is experimentally measured using a hydrophone without the presence of the test chamber. Its amplitude is then adjusted by applying an amplification factor obtained through a fitting procedure, matching the experimental and theoretical radius–time curves, to account for the variable sound absorption by nearby bubbles within the test chamber, as well as the acoustic reflections at the bubble’s location (Methods).

The time evolution of the vertical position of the microbubble centroid is also extracted from video recordings and compared with theoretical predictions (Fig. 2c). As the bubble displaces, it compresses the adjacent cell. For the first lower-amplitude ultrasound pulse, cell deformation is entirely reversible, as the bubble returns to its original position by the onset of the second pulse. Conversely, the second higher-amplitude pulse causes the cell to exceed its ultimate compression strain, creating a transendothelial tunnel. The bubble displacement is modelled through the force balance between the bubble inertia, the hydrodynamic forces, the cell resistive force and the acoustic driving forces (Methods). The acoustic driving forces include the primary Bjerknes force from the ultrasound and the secondary Bjerknes force from the rigid plastic substrate, viewed as a virtual bubble emitting a secondary sound field^44^. The contribution of the cell layer to the secondary Bjerknes force is negligible due to its softness, as is the buoyancy force due to the brief dynamics duration. Given the overall good agreement with experiments, this theoretical model, without free parameters, could be useful to effectively simulate the deformation of living tissue caused by ultrasound-driven microbubbles. Minor discrepancies observed during bubble compression phases can be attributed to the bubble collapse asymmetry, which is not accounted for in the spherically symmetric model.

In sonoporation studies, rigid plastic substrates are commonly used for growing endothelial cell monolayers because they substantially enhance cell proliferation, leading to a more uniform monolayer compared with soft substrates^45^. However, the influence on the bubble dynamics of a rigid plate beneath a soft material layer remains underexplored. Existing research on bubble dynamics involving such composite substrates is limited to numerical simulations conducted at high ultrasound pressures^46^. We assess this gap by comparing the effect of the plastic substrate against that of ultrasound, which is always present and serves as a baseline. By considering the pressure gradient from the ultrasound pulse and the pressure gradient from the sound field generated by the rigid backing plate (Fig. 2d), we compute the corresponding dimensionless impulses that these pressure gradients induce in the fluid during each ultrasound period. We extend the applicability of the dimensionless impulse, previously limited to collapsing vapour bubbles, to include bubbles subjected to a generic driving pressure (Methods). The impulse imparted by the plastic substrate surpasses that induced by ultrasound by a factor increasing from 3 to 15 as the bubble approaches the substrate (Fig. 2e). This suggests a potential influence on jet formation. To clarify this, we verify whether the jetting phenomenon persists when microbubbles interact with a thick soft substrate.

## Nature of jets

**Fig. 3:**
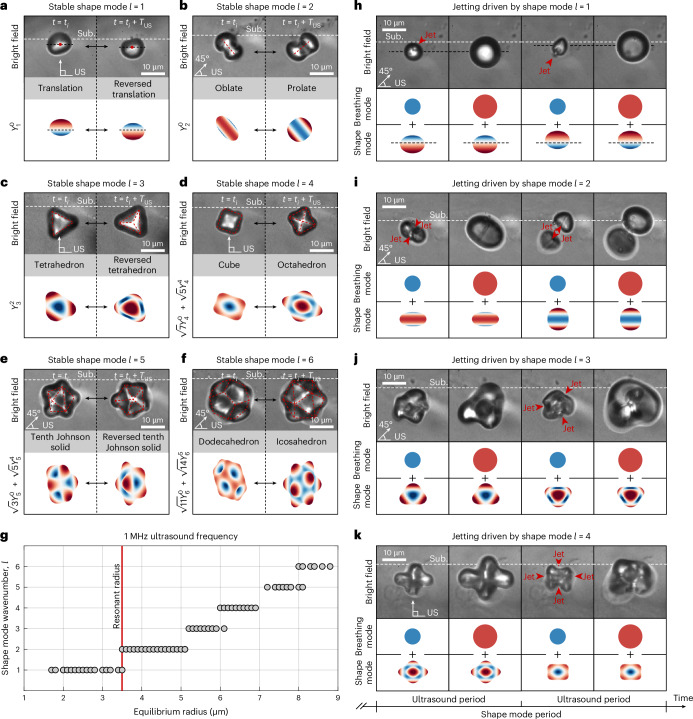
Shape modes and jet formation of single microbubbles in contact with a PEG substrate. **a**–**f**, Shape modes with angular wavenumbers *l* ranging from 1 to 6. The top panels display the bright-field snapshots from two consecutive ultrasound cycles, at a generic time instant *t* = *t*_*i*_ and one ultrasound (US) period *T*_US_ = 1 μs later, at *t* = *t*_*i*_ + *T*_US_. These images illustrate the cyclic transition between a geometric pattern and its dual during the shape mode oscillation. Odd-wavenumber shape mode patterns are self-dual. The red dashed sketches show the polyhedral representation of shape mode patterns. The bottom panels show the combination of spherical harmonics ${Y}_{l}^{\,m}$ representing the shape mode patterns. The red regions denote outward deformation, whereas the blue regions indicate inward deformation. **g**, Experimentally observed shape modes with angular wavenumber *l* as a function of the equilibrium bubble radius. The uncertainty in the bubble radius measurement corresponds to half the pixel size (80 nm). **h**–**k**, Jets driven by shape modes with angular wavenumbers *l* ranging from 1 to 4. The top panels show the bright-field images depicting the jets generated by the shape pattern or by its dual. The bottom panels show the bubble-shape decomposition in the breathing mode (spherical oscillation) and shape mode. The red regions denote outward deformation, whereas the blue regions indicate inward deformation. Jets manifest during compression phases in the sunken regions of the shape mode. Sub., substrate. Source data

As a thick soft substrate, we use a polyethylene glycol (PEG) hydrogel slab with a compression elastic modulus of *E* ≈ 0.5 kPa to mimic the softness of brain tissue^47^ (Methods). The optical properties of this test model are superior to those of the cellular substrate and, therefore, enable for a more comprehensive investigation of bubble dynamics. On ultrasound driving, we observe that the radial oscillation of the bubble destabilizes its interface once a threshold amplitude is exceeded, leading to the formation of a stable standing-wave pattern on the bubble surface, with a frequency half that of ultrasound driving. This phenomenon is driven by the Faraday instability^48^, which causes the appearance of half-harmonic patterns, known as shape modes, on oscillating density interfaces. We identify shape modes with angular wavenumbers *l* ranging from 1 to 6 (Fig. 3a–f and Supplementary Videos 4–9). The wavenumber increases with bubble size (Fig. 3g). A shape mode of wavenumber *l* can be expressed using spherical harmonics ${Y}_{l}^{m}$ (0 ≤ *m* ≤ *l*), where *l* and *m* denote its degree and order, respectively. The linear stability of the bubble interface is independent of the spherical harmonic order *m* (refs. ^49,50^). Consequently, each of the *l* + 1 possible *m* values is equally probable from a linear standpoint. The pattern that ultimately forms is, thus, a consequence of nonlinear effects. Specifically, for *l* = 1, we observe the bubble undergoing an alternating rigid-body motion (Fig. 3a). For *l* = 2, the shape mode oscillates between oblate and prolate shapes (Fig. 3b). For higher *l*, the shape mode alternates between polyhedral patterns and their duals, where the vertices of one correspond to the faces of the other and vice versa (Fig. 3c–f). Previous studies have already observed shape modes on bubbles^51,52^—including coated microbubbles^53–55^—but they were limited to reporting only the degree of the shape mode. By contrast, we reveal the full three-dimensional pattern of these shape oscillations and identify the combination of spherical harmonics that describe them (Fig. 3a–f). Our observations confirm past theoretical^56–59^ and numerical predictions^60^ concerning the dominant three-dimensional patterns of the Faraday instability on a spherical interface.

**Extended Data Fig. 3:**
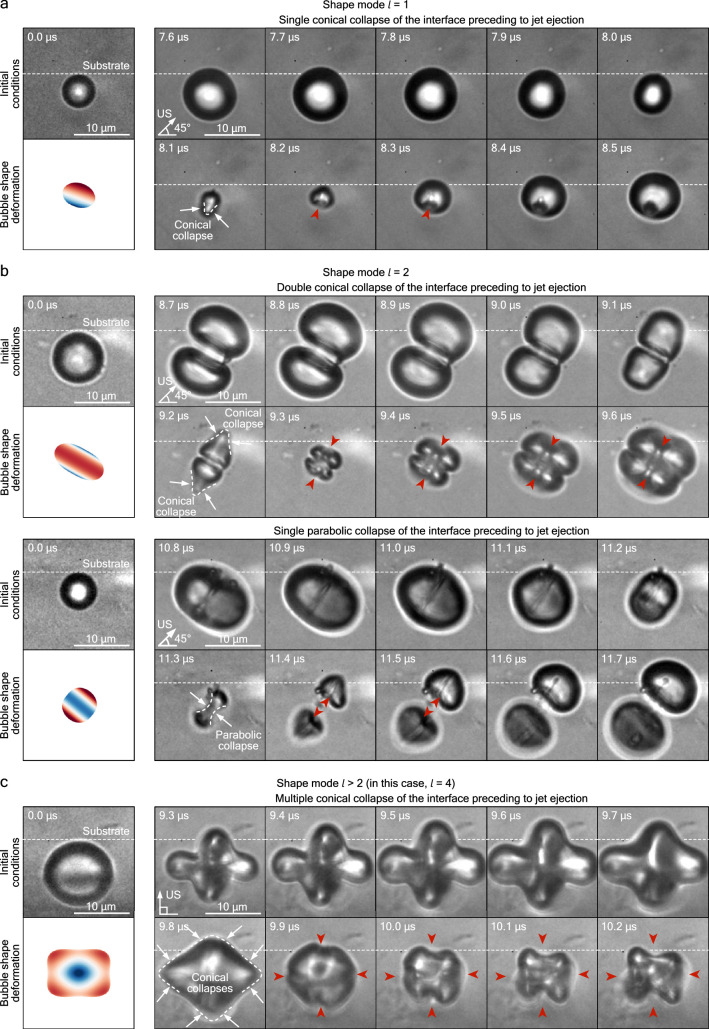
Shape of the bubble interface before jet emission. The red arrows indicate the jetting occurrence. (**a**) For the *l* = 1 shape mode, the single bubble lobe collapses taking a conical shape before ejecting a jet. (**b**) For the *l* = 2 shape mode, as the bubble transitions from a prolate to an oblate form, the two bubble lobes at opposite poles follow a conical collapse, releasing two converging jets. Conversely, when the bubble shifts from oblate to prolate, the equatorial region collapses annularly, adopting a parabolic profile. Two diverging jets are shoot up- and downwards from the closure point. (**c**) For *l* > 2 shape modes (*l* = 4 in the present case), the multiple bubble lobes undergo conical collapses, each producing a jet.

When the shape mode amplitude is sufficiently large, the shape lobes fold in rapidly during bubble compression and shape reversal, generating cyclic microjets (Fig. 3h–k and Supplementary Videos 10–13). We find that the number and orientation of the ejected jets depend on the specific shape mode. The *l* = 1 mode generates alternately directed single jets (Fig. 3h), whereas the *l* = 2 mode produces pairs of jets that alternately converge and diverge. (Fig. 3i). Higher wavenumbers *l* result in multiple jets that tend to match the number of faces of the polyhedral pattern (Fig. 3j,k). Shape-mode-induced cyclic jets on bubbles have probably been observed already in previous studies, which documented the occurrence of repeated jets resulting from surface deformation at driving frequencies spanning hertz^61^, kilohertz^62^ and even megahertz^63^ ranges. This jetting phenomenon can be considered as the spherical analogue to the jets formed by Faraday waves on vertically vibrating liquid baths^64,65^. Such jets emerge when the depressions created by Faraday waves undergo conical collapse. Although limited by the temporal resolution of the camera, we witness the same process occurring in our spherical interface scenario. For the *l* = 1 shape mode, the retracting lobe forms an approximately conical shape before ejecting a jet (Extended Data Fig. 3a). Jets arising from a collapsing conical interface are also observed in other problems, including a bubble bursting at fluid interfaces^66,67^, droplet impact on liquid pools^68,69^, cavitation bubble collapse in extremely close proximity to solid boundaries^70,71^ and coalescence of bubbles^72^. The *l* = 2 shape mode exhibits a more peculiar behaviour due to its axisymmetry (Extended Data Fig. 3b). When the bubble shifts from a prolate to an oblate form, the two retracting lobes at opposite poles become conical—similar to the *l* = 1 case—before emitting two jets that converge at the centre. Conversely, during the transition from oblate to prolate, the equatorial belt retracts annularly, adopting a parabolic profile. As it ruptures, two jets are ejected, diverging from the centre. The latter behaviour parallels Worthington jets observed when a solid impacts a liquid surface, generating an axisymmetric cavity that pinches off, producing jets directed upwards and downwards^73,74^. Both types of collapsing interface can be unified under a common theoretical framework for axisymmetric cavity collapse driven by a radial velocity field^75^. For shape modes with *l* > 2, the bubble produces multiple jets from its retracting lobes, each taking on a conical form (Extended Data Fig. 3c). Annular collapses with parabolic profiles are precluded, as modes with *l* > 2 are not axisymmetric. A follow-up study is currently underway to temporally resolve the interface shape that leads to jet formation and to provide a quantitative characterization.

**Extended Data Fig. 4:**
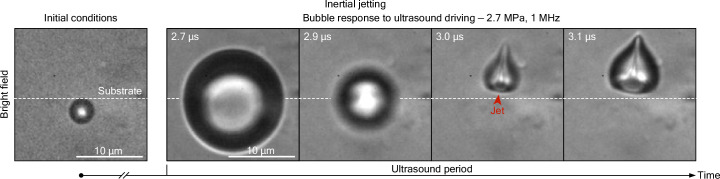
Inertial microjetting of a microbubble. Inertial jet generated by a microbubble (*R*_0_ = 2.3 *μ*m) in contact with a PEG hydrogel substrate, driven by a very intense ultrasound pulse (*p*_a_ = 2.7 MPa, *f* = 1 MHz). The jet is transient as it is followed by bubble fragmentation.

**Extended Data Fig. 5:**
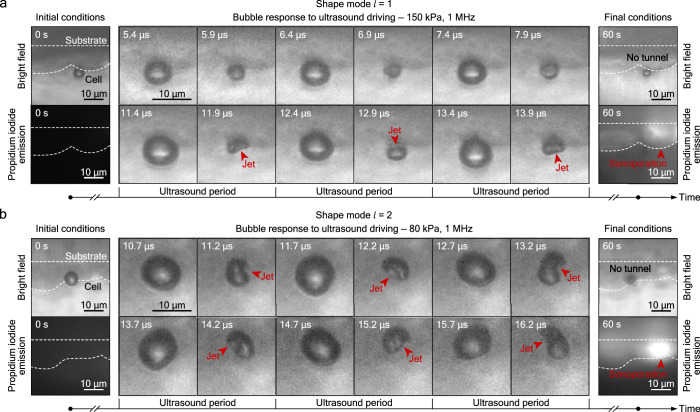
Shape modes drive cyclic microjetting on the cellular substrate, leading to cell membrane poration and drug uptake. (**a**) Jetting induced by a shape mode with wavenumber *l* = 1, as the bubble (*R*_0_ = 2.9 *μ*m) displays an alternate body motion (*p*_a_ = 150 kPa, *f* = 1 MHz). (**b**) Jetting induced by a shape mode with wavenumber *l* = 2, as the bubble (*R*_0_ = 3.8 *μ*m) alternately takes on a prolate and oblate shape (*p*_a_ = 80 kPa, *f* = 1 MHz).

Shape modes typically align one of their symmetry axes with the ultrasound direction (82% of 67 cases with ultrasound at a 90° angle to the substrate and 80% of 80 cases at a 45° angle). This ensures that at least one jet per shape mode period aligns with the ultrasound. For *l* = 1 and *l* = 2 modes, this results in the jet striking the substrate when the ultrasound is directed towards it. Although theoretically, modes with *l* ≥ 3 should not produce jets reaching the substrate as they converge at the bubble centre, deviations from the ideal shape often result in jets hitting the substrate. We conclude that cyclic jets directed against the substrate occur even without a rigid backing substrate. Consequently, these jets do not rely on a pressure-gradient driver to form but rather on interface instabilities, setting them apart from classical inertial jets^28^. Moreover, shape-mode-induced jets occur concurrently and repeatedly, unlike inertial jets, which are solitary and transient (Extended Data Fig. 4 and Supplementary Video 14). They also require approximately ten times lower acoustic pressures to initiate, as shape modes concentrate kinetic energy at single points on the bubble interface where the lobes fold inwards. The formation of shape-mode-induced jets remains consistent on the cellular substrate, too (Extended Data Fig. 5a,b and Supplementary Videos 15 and 16). Shape modes with *l* = 1 and *l* = 2 are clearly identified, whereas the higher modes are absent due to the radius of the bubbles studied being smaller than 5 μm. The first two modes are expected to be the most relevant for practical applications, as they manifest for bubble sizes at the sides of the resonant dimension, which, if targeted, allows to minimize the ultrasound pressure used (Fig. 3g). We note that the presence of a substantial pressure gradient, such as that caused by a neighbouring rigid substrate, can facilitate the formation of shape-mode-induced jets pointing away from the gradient and restraining those directed towards it (Fig. 1d).

## Inception threshold for jetting and sonoporation

**Fig. 4:**
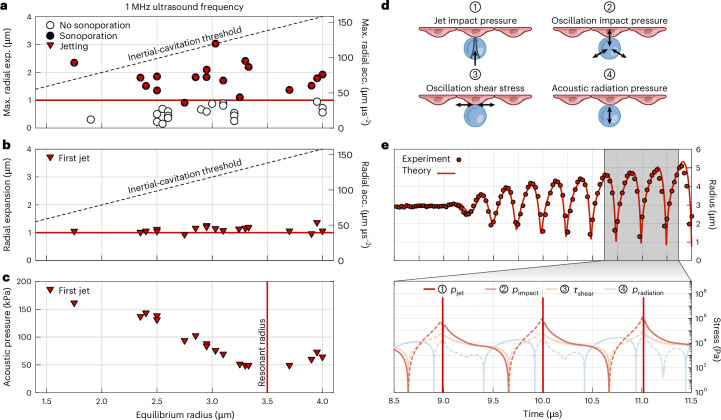
Cyclic microjetting as a mechanistic threshold indicator for sonoporation. **a**, Sonoporation outcome and jetting occurrence as a function of the bubble equilibrium radius, maximum radial expansion (Max. radial exp.) and corresponding maximum radial acceleration (Max. radial acc.). Cyclic microjetting and sonoporation both occur when the bubble expands beyond approximately 1 μm. The threshold for ‘inertial cavitation’, defined as when a bubble radially expands to twice its equilibrium radius, is also depicted. **b**, Appearance of the first microjet during the ultrasound pulse as a function of the bubble equilibrium radius, instantaneous radial expansion and corresponding instantaneous radial acceleration. The first microjet consistently forms when the bubble expands to at least 1 μm. **c**, Appearance of the first microjet during the ultrasound pulse as a function of the bubble equilibrium radius and the instantaneous ultrasound pressure amplitude. The minimum pressure required for jetting occurs when the bubble is at its resonant radius. The uncertainty in the bubble radius measurement corresponds to half the pixel size (80 nm). **d**, Different stress mechanisms elicited by an ultrasound-driven microbubble on a cell. **e**, Stress evolution over time in the logarithmic scale of each stress mechanism (bottom), inferred from the radial dynamics of the microbubble depicted in Fig. 1d and characterized in Fig. 2 (top). The solid lines represent stresses directed towards the substrate or outwards from the bubble, whereas the dashed lines indicate the opposite. Source data

Having elucidated the mechanism of jet formation, we now examine how bubble motion and jetting impact the cellular substrate and induce sonoporation. In our tests (*n* = 37), we observe a critical threshold for the maximum radial expansion of the bubble at around 1 μm, independent of the bubble equilibrium radius in the size range examined, beyond which cells experience sonoporation. In every instance in which sonoporation occurred (*n* = 19), the bubble produced cyclic microjets (Fig. 4a). The first microjet within an ultrasound pulse consistently appears when the bubble radial expansion reaches approximately 1 μm (Fig. 4b). These results suggest that sonoporation is enabled by microjetting. The ultrasound pressure required to exceed the radial expansion threshold for jetting depends on the bubble size and the driving frequency. For a driving frequency of 1 MHz, the necessary ultrasound pressure amplitude ranges from approximately 50 kPa for resonant-sized bubbles to around 200 kPa for non-resonant bubbles (Fig. 4c).

Prior studies have reported a similar critical threshold in bubble radial expansion for successful sonoporation when using microsecond-long ultrasound driving^30,38,40^. However, their top-view perspective on the cell monolayer did not reveal potential bubble jetting. Considering our analogous experimental conditions and outcomes, cyclic jetting could plausibly be the driving mechanism for sonoporation in those studies, too. The meaning of a threshold in radial expansion becomes clear when interpreted in terms of radial acceleration. Indeed, it is interfacial acceleration that destabilizes the surface between two fluids, possibly leading to jet formation. By approximating the interfacial acceleration as *a* ≈ (*R*_max_ − *R*_0_)*ω*^2^, where *R*_max_ – *R*_0_ is the maximum bubble expansion and *ω* is the angular driving frequency, we identify a threshold in acceleration for jetting at around 40 μm μs^−2^ (Fig. 4a,b).

Common beliefs associate bubble jetting solely with ‘inertial cavitation’, a regime characterized by violent bubble collapse and fragmentation, typically occurring when the bubble expands beyond twice its equilibrium size. However, the cyclic jetting observed here, which results from interface instabilities, predominantly occurs within the ‘stable-cavitation’ regime, where bubbles exhibit a relatively gentle response (Fig. 4a,b).

## Jetting stress evaluation

Besides microjetting, the bubble can cause mechanical damage to the neighbouring cell layer through several other mechanisms. These include repeated collisions caused by the bubble oscillation, alternating viscous stresses from the oscillatory flow field, steady viscous stresses arising from acoustic streaming and penetrating stress exerted by the bubble propelled by Bjerknes forces^9^ (Fig. 4d). To understand why microjetting is the mechanism enabling sonoporation among all others, we quantify and compare the stress over time induced by each mechanism (Fig. 4e), inferred from the observed bubble motion in the successful sonoporation test case depicted in Fig. 1d and characterized in Fig. 2 (Methods).

The resulting magnitudes of the normal and shear stresses are approximately consistent with previously reported values obtained from three-dimensional finite-element-method simulations^76,77^. The jets produced by the bubble are observed to traverse the entire bubble and impact the substrate within a single frame (0.1 μs), suggesting a jet velocity *u*_jet_ > 60 ms^−1^. A more accurate measurement is beyond our current experimental capabilities. At these jet speeds, the resultant jet hammer pressure exceeds that of any other mechanism by at least 30-fold. However, this elevated pressure is sustained only briefly, persisting only for the time it takes for a rarefaction wave, generated at the jet contact edge, to reach its central axis. This duration can be estimated, considering the jet head as spherical^78^, as *ς* ≈ *R*_jet_*u*_jet_/*c*^2^ > 20 ps, where *R*_jet_ represents the jet radius. Subsequently, the pressure along the central axis declines to the lower Bernoulli stagnation pressure, which remains more than fourfold higher than the pressure generated by the side of the bubble impacting the cell. Furthermore, the pressure exerted by the jet hammer is concentrated over a smaller area compared with other sources of stress^78^. The radius of the contact area can be approximated as *ϱ* ≈ *R*_jet_*u*_jet_/*c* > 30 nm. This value aligns with the pore sizes reported in previous studies that used similar ultrasound driving parameters^29,79^. The substantially higher stress generated by the jet on impact can explain why, for microsecond-long ultrasound driving, only jets enable sonoporation. However, we cannot rule out the possibility that extended exposure to ultrasound—lasting several milliseconds, as sometimes used in other sonoporation studies^80^—may enable weaker stress sources to induce sonoporation.

In conclusion, our study addresses several open questions related to the physics behind drug delivery mediated by microbubbles. We have elucidated the mechanism of action—cyclic bubble jetting—and the physics behind its formation, modelled bubble and cell dynamics, estimated the mechanical stress generated and defined thresholds pertinent to bubble dynamics for successful drug delivery. These findings are expected to substantially advance the development of microbubble-mediated drug delivery systems, accelerating their translation into clinical practice. Beyond biomedical treatments, the impact of this study extends to diverse technological fields involving bubbles and acoustics, such as sonochemistry, additive manufacturing and advanced cleaning technologies.

## Methods

### Cell monolayer culture

**Extended Data Fig. 6:**
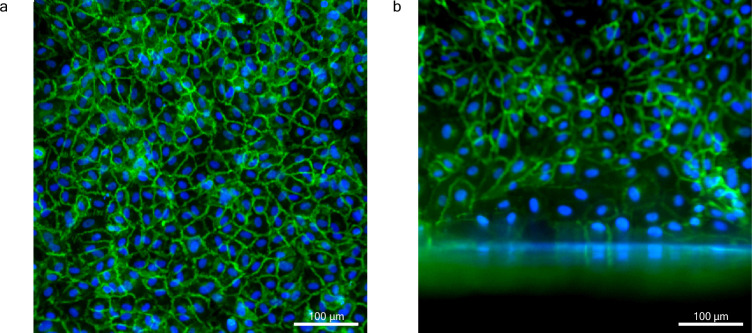
Fluorescence images of human umbilical vein endothelial cells grown on a plastic substrate. Confluence of the cell monolayer (**a**) in the center and (**b**) at the edge of the membrane. Cells are stained with DAPI (blue) and CD31 antibody (green). The images are representative of three independently prepared monolayers, each created on a separate day (n=3 biological replicates).

Primary HUVECs (cat. no. C-12200, PromoCell) are cultured in an endothelial growth medium (EGM-2, Lonza Bioscience AG) supplemented with 10% foetal bovine serum (Gibco) within collagen-coated (Corning Collagen I, rat tail) T75 flasks at 37 °C in a humidified incubator with 5% CO_2_. The medium is changed every three days and cells are passaged before reaching full confluence. The HUVECs used are below passage 12. Cells are detached using 0.05% Trypsin-EDTA (Gibco) and seeded on collagen-coated polycarbonate membranes (thickness, 50 μm; surface area, 1 cm^2^) cut from a CLINIcell (MABIO). The membranes are then incubated for four days to form confluent cell monolayers, with the medium being replaced on the third day. An example of the resulting cell monolayer is shown in Extended Data Fig. 6. For imaging it, the cell monolayer is fixed with 4% paraformaldehyde (Artechemis), washed with PBS (1×, Gibco) and incubated for 30 min at room temperature in 1% bovine serum albumin (AppliChem) and 0.3% Triton X-100 (Sigma-Aldrich) in PBS. Cell junction staining is then performed by incubation with a mouse anti-CD31 primary antibody (cat. no. 555444, monoclonal, clone WM59, BD Biosciences) at 1:200 dilution overnight at 4 °C. Consequently, cells are washed with PBS for 3 h and incubated with Alexa Fluor 488 goat-anti-mouse secondary antibody (cat. no. ab150113, polyclonal, abcam) at 1:200 dilution and 4′,6-diamidino-2-phenilindol (Sigma-Aldrich) at 1 μg ml^−1^ for nuclei staining overnight at 4 °C. HUVECs are then washed for 3 h and imaged with a Leica DMI6000B inverted epifluorescence microscope.

### Microbubble preparation

Lipid-coated microbubbles are prepared in-house by probe sonication following our previously described protocol^81^ (Supplementary Information). The gas core is made of perfluorobutane (Fluoromed) and the lipid coating consists of 90 mol% of 1,2-distearoyl-*sn*-glycero-3-phosphocholine (NOF EUROPE) and 10 mol% of 1,2-distearoyl-*sn*-glycero-3-phosphoethanolamine-N-[methoxy(polyethylene glycol)-2000] (Larodan).

### Test chamber preparation

A 10 × 10 × 10 mm^3^ water-tight test chamber is used to enclose the cell monolayer (Fig. 1b). The chamber features transparent acrylic side walls and is open at both top and bottom. A silicon rubber cap seals one aperture. The plastic substrate cultivated with cells is placed in the chamber in contact with the rubber cap. The chamber is filled with a solution containing PBS 1×, PI (Sigma-Aldrich, 25 μg ml^−1^) and microbubbles (~500 microbubbles ml^–1^). Tape seals the second opening, still allowing the transmission of the ultrasound pulse.

### PEG hydrogel substrate preparation

PEG hydrogels are prepared following previously described protocols^82,83^ (Supplementary Information). For the measurement of the compression elastic modulus of PEG hydrogels via atomic force microscopy (AFM), 10 μl gel samples are fabricated between two hydrophobic glass slides, separated by a 0.5 mm rubber spacer to ensure a flat topography. For the experiments with microbubbles, the hydrogel precursor solution is placed on one of the external surfaces of a square glass capillary with a cross-section of 0.90 × 0.90 mm^2^ (CM Scientific), which has been pretreated with plasma. The capillary serves as a support for the hydrogel substrate during the experiments. To maintain the solution atop the capillary during gelation, two rubber delimiters are used. The resulting thickness of the hydrogel substrate is 2 mm.

### Experimental setup

The cell monolayer test chamber, or alternatively, the capillary holding the PEG substrate, is immersed in a water bath filled with deionized water (*T*_l_ ≈ 22 °C). When using the PEG substrate, microbubbles are introduced using a syringe beneath the substrate, where they adhere via flotation. To mitigate potential interference, the microbubbles under examination are situated at least 50 μm away from the walls of the test chamber or the periphery of the PEG substrate. For gaining comprehensive insights into the behaviour of single microbubbles interacting with cells and to evaluate the resulting sonoporation outcome, we developed a horizontal microscope (Extended Data Fig. 1), which enables ultrahigh-speed bright-field and fluorescence recordings of the monolayer with a side-view perspective. The system is realized using modular optomechanics components (Thorlabs, cage system) and installed on an optical table (T1220C, Thorlabs) with active isolators (PTS603, Thorlabs) to minimize environmental vibrations. The microscope features a water-dipping objective lens (CFI Plan 100XC W, Nikon) with a focal length of 2 mm and a tube lens (TL400-A, Thorlabs) with a focal length of 400 mm, resulting in a total magnification of ×200. The terminal section of the microscope, housing the objective lens, is inserted in the water bath through a sealed opening in the water container. An ultrahigh-speed camera (HPV-X2, Shimadzu) allows for recordings at 10 million frames per second of a 64 μm × 40 μm field of view with a 160 nm pixel resolution. Backlight illumination is provided by a continuous halogen illuminator (OSL2, Thorlabs) and two xenon flash lamps operated sequentially (MVS-7010, EG&G), dedicated for live imaging and video recording, respectively. The flash lamps provide sufficient illumination for about 20 μs of recording. All the light sources are combined into a single optical-fibre output and focused on the sample with a custom-built condenser (L1 and L2, AC127-025-A, Thorlabs). The test chamber or the capillary position is controlled by a three-axis motorized microtranslation stage (PT3/M-Z8, Thorlabs).

A high-intensity focused ultrasound transducer (PA1280, Precision Acoustics; centre frequency, 1 MHz; focal length, 75 mm; beamwidth, ~3 mm; –6 dB) is used to drive the microbubbles. When the test chamber is used, the transducer is positioned in the water bath at an angle of 75° with respect to the horizontal plane to minimize acoustic reflections within the test chamber. Conversely, when the capillary is used, the transducer is positioned at either a 90° or 45° angle. The driving pulse is generated with a function generator (LW420B, Teledyne LeCroy) and amplified by a radio-frequency power amplifier (1020L, E&I). A calibrated needle hydrophone (0.2 mm, NH0200, Precision Acoustics) is used to align the ultrasound focal point with the optical field and to record the shape of the ultrasound pulse envelope, which is utilized as input for the bubble radial dynamics model. The transducer is manoeuvred using a manual three-axis microtranslation stage (three units of DTS50/M, Thorlabs).

Fluorescence microscopy is conducted by using a 532 nm continuous-wave laser (Verdi G10, Coherent) as the excitation light source. An acousto-optic tunable filter (AOTF.NC-VIS/TN, AA Opto-Electronic) acts as an electronic laser shutter. The transmitted beam (zeroth order) ceases, whereas the diffracted beam (first order) can be activated or deactivated by adjusting the radio-frequency drive power supplied by the acousto-optic tunable filter driver (MOD.8C.10.b.VIS, AA Opto-Electronic). To achieve a laser spot size matching the entire field of view, the beam is enlarged by using a 10× beam expander (52-71-10X-532/1064, Special Optics). The laser beam is then focused on the back focal plane of the objective lens by adjusting the spacing of a lens relay system (L3 and L4, AC254-100-A, Thorlabs) to achieve a collimated beam emerging from the objective lens. Undesirable light wavelengths are blocked from reaching the specimen by means of a narrow passband excitation filter (ZET532/10×, Chroma). The laser beam is steered into the objective lens using a reflective-band dichroic beamsplitter (ZT532dcrb, Chroma). The laser line is removed from the specimen image with an emission filter (ET590/50m, Chroma).

The activation of the ultrasound pulse, camera recording, light flash and laser switching are synchronized through a delay generator (DG645, Stanford Research Systems).

### AFM measurements of the compression elastic modulus of PEG hydrogel substrates

**Extended Data Fig. 7:**
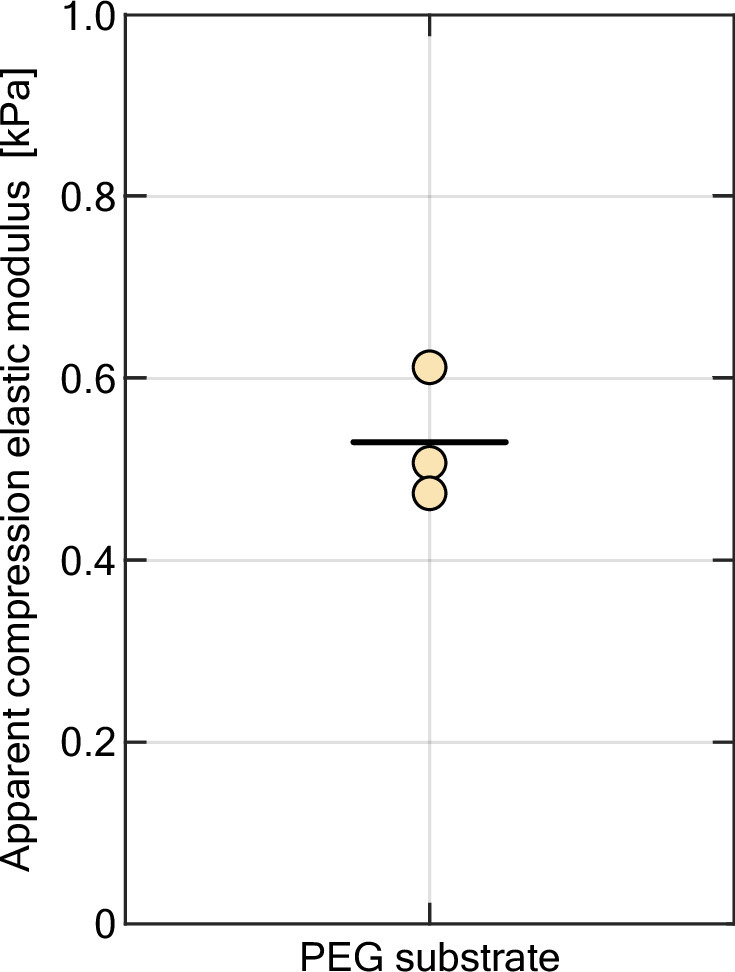
Apparent compression elastic modulus of PEG hydrogel samples measured by atomic force microscopy. Each data point represents the average of median values of the apparent elastic modulus measured at four locations per hydrogel sample, with each location sampled on a 5 × 5 points force map. Measurements are performed on three independently prepared hydrogel samples, with each sample fabricated and tested on different days (n = 3 biological replicates). Source data

PEG hydrogels meant for AFM-based mechanical characterization are placed on positively charged glass slides (Superfrost, Thermo Fisher Scientific) for improved adhesion and secured attachment during measurements. A hydrophobic marker pen is used to trace the area surrounding the hydrogel and 300–400 μl of PBS at room temperature is used to fully immerse the gel, ensuring hydration during the indentation measurements. Nanoindentation measurements are performed using a Flex-Bio AFM instrument (Nanosurf). The hydrogel surface is indented using a colloidal probe made up of a cantilever with a nominal spring constant of *k* = 0.1 N m and a 10-μm-diameter borosilicate glass bead affixed to the cantilever tip (CP-qp-CONT-BSG-B-5, Nanosensors). Before testing, the spring constant is calculated using the Sader method implemented in the Nanosurf Flex-Bio control software (Nanosurf C3000 version 3.10.0.26). The slope of the deflection–displacement curve, obtained from the indentation of a bare region of the glass slide, is used to determine the deflection sensitivity. The AFM is mounted on top of an inverted microscope (Eclipse Ti-E, Nikon) to allow for sample visualization and macroscopic positioning of the probe. The deflection and displacement of the cantilever are recorded as the probe descends, indenting the gel surface at a speed of 1 μm s^−1^ and then retracts, producing a force–displacement curve for each indentation. Between four and five random locations, each measuring 50 × 50 μm^2^, are selected for indentation on each sample. At each location, measurements are taken at a grid of 5 × 5 points. To obtain the apparent compression elastic modulus *E* from each force–displacement curve, and in accordance with current standard practices in AFM nanoindentation, we use the Hertz contact model, which is considered to be the most appropriate for a sphere indenting a semi-infinite half-space:

1 $$F=\frac{4}{3}\frac{E}{1-{\vartheta }^{2}}{{\mathcal{R}}{}^{1/2}\iota }^{3/2},$$

where *F* is the force applied by the cantilever, *ϑ* is the Poisson’s ratio, ${\mathcal{R}}$ is the radius of the spherical bead and *ι* is the indentation. For consistency with the literature and simplification, the Poisson’s ratio is considered to be 0.5. Each curve is fitted using a custom-built Python-based algorithm (Python 3.11) to extract the apparent elastic modulus. Force–displacement curves without a clear contact point are discarded. The mean apparent compression elastic modulus of PEG hydrogels is *E* = 0.53 ± 0.07 kPa (Extended Data Fig. 7). This value is obtained by averaging the mean values of the median apparent modulus values in 4–5 locations in each hydrogel (*n* = 3).

### Image analysis

The time evolution of the microbubble radius and position is extracted from the bright-field high-speed recordings using a feature extraction script written in MATLAB R2023a (MathWorks; Supplementary Information).

### Theoretical modelling of bubble radial dynamics

The radial motion of a coated microbubble, assumed spherical with radius *R*(*t*), is described using a Rayleigh–Plesset-type model that has been introduced in our earlier study^81^ (Supplementary Information).

### Theoretical modelling of bubble displacement dynamics

A microbubble, assumed spherical with radius *R*(*t*), in contact with a soft substrate and driven by an ultrasound pulse directed normal to the substrate, displays a translational motion *x*(*t*) (assumed positive if towards the substrate) that can be described using the following force balance equation:

2 $$\underbrace{{F}_{{\rm{I}}}(t)}_{{\rm{Inertial}}\,{\rm{force}}}=\quad \,\underbrace{{F}_{{\rm{B1}}}(t)+{F}_{{\rm{B2}}}(t)}_{{\rm{Acoustic}}\,{\rm{driving}}\,{\rm{forces}}}\quad \,+\,\,\quad \underbrace{{F}_{{\rm{AM}}}(t)+{F}_{{\rm{VD}}}(t)}_{{\rm{Hydrodynamic}}\,{\rm{forces}}}\quad \,\,+\underbrace{{F}_{{\rm{CR}}}(t).}_{{\rm{Cell}}\,{\rm{resistive}}\,{\rm{force}}}$$

*F*_I_(*t*) is the inertial force of the bubble, characterized by its mass *m*:

3 $${F}_{{\rm{I}}}(t)=m\ddot{x}.$$

*F*_B1_(*t*) is the primary Bjerknes force induced by the ultrasound driving pressure gradient $\frac{\partial p_{\mathrm{d}}}{\partial x}$ on a bubble of volume *V* (ref. ^84^):

4 $${F}_{{\rm{B1}}}(t)=-V\,\frac{\partial p_{\mathrm{d}}}{\partial x}.$$

*F*_B2_(*t*) is the secondary Bjerknes force caused by the presence of a rigid plastic substrate at a distance *L* – *x*, where *L* is the initial thickness of the cell^44^. In accordance with the potential flow theory, this plastic substrate can be represented by a virtual bubble that mirrors the real one and, therefore, emits a sound field:

5 $${F}_{{\rm{B2}}}(t)=-\frac{{\rho }_{{\rm{l}}}V}{16\uppi {(L-x)}^{2}}\ddot{V},$$

where *ρ*_l_ is the liquid density. In the example reported in Fig. 1, the cell thickness amounts to *L* ≈ 12 μm. *F*_AM_(*t*) is the added mass force that accounts for the additional fluid mass that gets carried along by the bubble as it moves through the fluid^85^:

6 $${F}_{{\rm{AM}}}(t)=-\frac{1}{2}{\rho }_{{\rm{l}}}\left(\dot{V}x+V\dot{x}\right).$$

*F*_VD_(*t*) is the quasi-steady viscous drag force experienced by the translating bubble^85^:

7 $${F}_{{\rm{VD}}}(t)=-\frac{1}{2}{\rho }_{{\rm{l}}}\uppi {R}^{2}{\dot{x}}^{2}{C}_{{\rm{D}}},$$

where the drag coefficient *C*_D_ is taken as^86^

8 $${C}_{{\rm{D}}}=\frac{24}{{\rm{Re}}}+\frac{6}{1+\sqrt{{\rm{Re}}}}+0.4,$$

with $\,\text{Re}\,=2{\rho }_{{\rm{l}}}R\dot{x}/{\mu }_{{\rm{l}}}$ denoting the instantaneous value of the translational Reynolds number, where *μ*_l_ is the fluid viscosity. The history contribution of the viscous drag force can be neglected at the time-averaged Reynolds numbers, $\widetilde{\,\text{Re}\,}$ and $\widetilde{{\mathcal{U}}\,\text{Re}\,}$, with ${\mathcal{U}}=\dot{R}/\dot{x}$, encountered in the experiments ($\widetilde{\,\text{Re}\,}=0.9$ and $\widetilde{{\mathcal{U}}\,\text{Re}\,}=16.5$ for the one reported in Fig. 1c and $\widetilde{\,\text{Re}\,}=4.3$ and $\widetilde{{\mathcal{U}}\,\text{Re}\,}=32.7$ for the one reported in Fig. 1d)^87^.

*F*_CR_(*t*) is the cell resistive force:

9 $${F}_{{\rm{CR}}}(t)=-\uppi {R}^{2}{c}_{\beta }\frac{{\text{d}}^{\beta }\epsilon }{\text{d}\,{t}^{\beta }},$$

where *ϵ* = *x*/*L* is the cell strain. Here we use a single fractional unit, also called a spring pot, to characterize the rheological behaviour of the living cell^88^. A spring pot leverages the concept of fractional derivative to capture behaviours that lie between those of a spring (*β* = 0) and a dash-pot (*β* = 1). Our approach is inspired by recent validated models for living cells that use two parallel fractional units to describe the different behaviours at low and high deformation rates^89^. We simplify this framework by only using the fractional unit associated with high deformation rates as the dynamics under consideration involve exceptionally rapid deformation (10^5^–10^7^ s^−1^). On the basis of microrheological studies performed on epithelial cells^89^, we adopt *β* = 0.8 and *c*_*β*_ = 1 Pa s^*β*^. A fractional derivative of order *β* with respect to time can be defined according to the Caputo approach^90^ as

10 $$\frac{{\text{d}}^{\beta }\epsilon (t)}{\text{d}{t}^{\beta }}=\frac{1}{\varGamma (n-\beta )}\mathop{\int}\nolimits_{0}^{t}{(t-s)}^{(n-\beta -1)}\frac{{\text{d}}^{n}\epsilon (s)}{\text{d}{s}^{n}}\,\,\text{d}s,\quad n-1 < \beta < n,$$

where *n* is an integer number and *Γ* is the gamma function. For 0 < *β* < 1, a simplified expression can be derived as

11 $$\frac{{\text{d}}^{\beta }\epsilon (t)}{\text{d}{t}^{\beta }}=\frac{1}{\varGamma (1-\beta )}\mathop{\int}\nolimits_{0}^{t}{(t-s)}^{(-\beta )}\frac{\text{d}\epsilon (s)}{\text{d}s}\,\,\text{d}s,\quad 0 < \beta < 1,$$

We use the L1 discretization^91^ to numerically compute the Caputo derivative at discrete time points *t*_*j*_ = *jτ*, with *j* = 0, 1, 2…*N*:

12 $${\left.\frac{{\text{d}}^{\beta }\epsilon (t)}{\text{d}{t}^{\beta }}\right\vert }_{{t}_{k}}=\frac{{\tau }^{-\beta }}{\varGamma (2-\beta )}\mathop{\sum }\limits_{j=1}^{k}{b}_{k-j}\left(\epsilon ({t}_{j})-\epsilon ({t}_{j-1})\right),$$

where *b*_*k*−*j*_ = (*k* – *j* + 1)^1−*β*^ – (*k* – *j*)^1−*β*^.

The gravity effects are disregarded in the force balance due to their negligible contribution.

### Derivation of a dimensionless impulse for ultrasound-driven bubbles

A bubble, initially at rest in an aqueous medium (of radius *R*_0_ and internal pressure *p*_g_ + *p*_v_, where *p*_g_ is the gas pressure and *p*_v_ is the vapour pressure, exposed to a smooth pressure field *p*(**x**), described by the approximation *p*(**x**) = *p*_0_ + 𝛁*p* ⋅ **x** + *O*(**x**^2^) in the vicinity of the bubble), develops a jet directed inwards against the local pressure gradient 𝛁*p* when the driving pressure jump is Δ*p* = *p*_0_ − *p*_g_ − *p*_v_ + 2*σ*_0_/*R*_0_ > 0, where *σ*_0_ is the surface tension.

It has been shown^92^ that for collapsing laser-induced millimetric vapour bubbles exposed to a uniform gravity-induced pressure gradient, the normalized jet volume correlates with the pressure anisotropy parameter **ζ**, defined as

13 $${\bf{\zeta }}=-{\boldsymbol{\nabla}}p{R}_{0}/\Delta p.$$

In this context, 𝛁*p* = *ρ*_l_**g**, where **g** is the gravitational acceleration and *R*_0_ stands for the bubble radius at the onset of collapse. Also, Δ*p* = *p*_0_ − *p*_v_ as the surface tension effects are neglected due to the inertia-dominated dynamics. **ζ** can be regarded as the dimensionless counterpart of the Kelvin impulse **I**, which measures the linear momentum acquired by the fluid during the expansion and subsequent compression phase of the bubble:

14 $${\bf{I}}=-{\int}_{\!\!T}{\int}_{\!\!S}p({\bf{x}}){\hat{\bf{n}}}\,{\rm{d}}S\,{\rm{d}}t\simeq -{\int}_{\!\!T}V{\boldsymbol{\nabla }}p\,{\rm{d}}t,$$

where *T* is the expansion and compression time period, *S* is the bubble surface and ${\hat{\boldsymbol{n}}}$ is the outward normal to the fluid. For a collapsing vapour bubble subjected to a constant pressure gradient (such as that induced by gravity), the Kelvin impulse **I** reads

15 $${\bf{I}}\simeq -4.789{R}_{0}^{4}{\boldsymbol{\nabla }}p\sqrt{\rho_{\rm l} /\Delta p},$$

and it is, therefore, linked to **ζ** through the relation:

16 $${\bf{I}}\simeq 4.789{R}_{0}^{3}\sqrt{\rho_{\rm l} \Delta p}{\bf{\zeta }}.$$

Another work^93^ experimentally found that **ζ** governs numerous other dimensionless jet parameters of collapsing vapour bubbles via power laws. Furthermore, this holds true irrespective of the type of jet driving, namely, gravity, free boundaries, rigid boundaries or any combination thereof. However, in the presence of a boundary, the pressure gradient 𝛁*p*(*t*) varies in time during the growth and collapse of a bubble, making it challenging to define **ζ** as an integral quantity akin to the Kelvin impulse **I**. Therefore, Supponen et al.^93^ defined an equivalent **ζ** for neighbouring boundaries such that equation (16) still returns the correct Kelvin impulse.

For ultrasound-driven bubbles, both pressure gradient 𝛁*p*(*t*) and driving pressure jump Δ*p*(*t*) vary in time. Hence, we cannot directly derive the value of **ζ** from equation (16) due to the dependence of the prefactor on a time-invariant Δ*p*. To proceed, we need to establish an equivalent time-invariant $\overline{{\Delta }p}$ for every ultrasound cycle to align ourselves with the framework in which equation (16) was derived. We can do this by recognizing that the driving pressure jump Δ*p*(*t*) is unaffected by the existing pressure gradient 𝛁*p*(*t*), enabling us to effortlessly determine it from the virtual impulse ${\hat{\bf{I}}}$ generated by a discretionary pressure gradient of constant known value, even unitary $\widehat{\boldsymbol{\nabla} p}$ (by means of equation (15)), as follows:

17 $${\overline{\Delta}}p={(4.789{R}_{0}^{4}{\widehat{\boldsymbol{\nabla}p}}\sqrt{\rho_{\rm{l}} }/{\hat{{\bf{I}}}})}^{2},$$

where *R*_0_ is the largest radius reached by the bubble during the ultrasound cycle, akin to the definition of *R*_0_ for a collapsing vapour bubble. At this point, **ζ** can be promptly calculated using the approach described in ref. ^93^, by deriving it from the actual impulse acquired by the fluid within the ultrasound cycle **I** by means of equation (16), as follows:

18 $${\bf{\zeta }}\simeq {\bf{I}}/4.789{R}_{0}^{3}\sqrt{\rho_{\rm l} \overline{{\Delta }p}}.$$

### Stress estimation of bubble damage mechanisms

On impact, a fluid jet induces a localized and transient high-pressure region on the cell. For a jet travelling at speed *u*_jet_ impacting a substrate with density *ρ*_l_ and speed of sound *c*_l_ comparable to water, the generated pressure can be evaluated using the water hammer pressure formula^94,95^:

19 $${p}_{{\rm{jet}}}\approx \frac{1}{2}{\rho }_{{\rm{l}}}{c}_{{\rm{l}}}{u}_{{\rm{jet}}}.$$

The impact (or suction) pressure exerted on the cell by a bubble expanding (or contracting) at rate $\dot{R}$ can be estimated using the Bernoulli stagnation pressure:

20 $${p}_{{\rm{impact}}}(t)\approx \frac{1}{2}{\rho }_{{\rm{l}}}| \dot{R}| \dot{R}.$$

The tangential viscous stress acting on the cell from the oscillatory flow field can be approximated from the quotient of the bubble wall velocity $\dot{R}$ and the boundary layer thickness *δ* (ref. ^96^):

21 $${\tau }_{{\rm{shear}}}(t)\approx {\mu }_{{\rm{l}}}\frac{\dot{R}}{\delta },$$

where *δ* can be determined as *δ* = (2*μ*_l_/*ρ*_l_*ω*)^1/2^.

The oscillatory flow field produces a secondary steady flow known as acoustic streaming. This motion, being a second-order effect, is typically 1–2 orders of magnitude weaker than the primary oscillatory flow field^97^. For coated microbubbles exposed to ultrasound pressures similar to those used in this study, the maximum observed acoustic streaming velocity does not exceed *u*_stream_ ≈ 0.1 m s^−1^ (refs. ^98,99^). Consequently, the resulting stress, that is,

22 $${\tau }_{{\rm{stream}}}\approx {\mu }_{{\rm{l}}}\frac{{u}_{{\rm{stream}}}}{\delta },$$

remains below a few hundreds of pascals. Moreover, acoustic streaming initiates only after several cycles of bubble oscillation and can, thus, be neglected in scenarios involving ultrasound pulses lasting mere tens of microseconds, as in our study.

Finally, the primary and secondary Bjerknes forces generate an average pressure on the bubble cross-section^44,84^, expressed as

23 $${p}_{{\rm{B1}}}(t)=-\frac{4}{3}R\frac{\partial p_{\mathrm{d}}}{\partial x}$$

for the primary Bjerknes force and

24 $${p}_{{\rm{B2}}}(t)=-\frac{1}{12\uppi {h}^{2}}{\rho }_{{\rm{l}}}R\ddot{V}$$

for the secondary Bjerknes force, where *h* is the distance between the bubble and plastic substrate.

### Reporting summary

Further information on research design is available in the Nature Portfolio Reporting Summary linked to this article.

## Online content

Any methods, additional references, Nature Portfolio reporting summaries, source data, extended data, supplementary information, acknowledgements, peer review information; details of author contributions and competing interests; and statements of data and code availability are available at 10.1038/s41567-025-02785-0.

## Supplementary information

Supplementary Information Supplementary Methods and Description of supplementary videos.

Reporting Summary

Supplementary Video 1 Response of a 3-μm-radius microbubble in contact with an endothelial cell to an ultrasound pulse (*f* = 1 MHz, *p*_a_ = 60 kPa, 20 cycles), captured from a side-view perspective. The amplitude of the applied ultrasound pressure is insufficient to induce the formation of cyclic piercing microjets from the microbubble. The field of view is 40 × 40 μm^2^. This video corresponds to the image sequence shown in Fig. 1c.

Supplementary Video 2 Response of the same 3-μm-radius microbubble as that in Supplementary Video 1 in contact with an endothelial cell to a more intense ultrasound pulse (*f* = 1 MHz, *p*_a_ = 160 kPa, 20 cycles), captured from a side-view perspective. The higher amplitude of the applied ultrasound pressure leads to the formation of cyclic piercing microjets directed towards the cell, resulting in cell membrane poration and drug uptake. The bubble motion also results in the formation of a transendothelial tunnel. The field of view is 40 × 40 μm^2^ and the recording speed is 10 million frames per second. This video corresponds to the image sequence shown in Fig. 1d.

Supplementary Video 3 Response of a 3-μm-radius microbubble in contact with an endothelial cell to an ultrasound pulse (*f* = 1 MHz, *p*_a_ = 175 kPa, 20 cycles), captured from a side-view perspective. In this instance, the bubble generates cyclic piercing microjets that facilitate drug uptake, but the motion of the bubble does not result in the formation of a transendothelial tunnel. The field of view is 40 × 40 μm^2^ and the recording speed is 10 million frames per second. This video corresponds to the image sequence shown in Extended Data Fig. 2.

Supplementary Video 4 Ultrasound-driven microbubble exhibiting a shape mode with an angular wavenumber *l* = 1. The field of view is 40 × 40 μm^2^ and the recording speed is 10 million frames per second. This video corresponds to the image sequence shown in Extended Data Fig. 3a.

Supplementary Video 5 Ultrasound-driven microbubble exhibiting a shape mode with an angular wavenumber *l* = 2. The field of view is 40 × 40 μm^2^ and the recording speed is 10 million frames per second. This video corresponds to the image sequence shown in Extended Data Fig. 3b.

Supplementary Video 6 Ultrasound-driven microbubble exhibiting a shape mode with an angular wavenumber *l* = 3. The field of view is 40 × 40 μm^2^ and the recording speed is 10 million frames per second. This video corresponds to the image sequence shown in Extended Data Fig. 3c.

Supplementary Video 7 Ultrasound-driven microbubble exhibiting a shape mode with an angular wavenumber *l* = 4. The field of view is 40 × 40 μm^2^ and the recording speed is 10 million frames per second. This video corresponds to the image sequence shown in Extended Data Fig. 3d.

Supplementary Video 8 Ultrasound-driven microbubble exhibiting a shape mode with an angular wavenumber *l* = 5. The field of view is 40 × 40 μm^2^ and the recording speed is 10 million frames per second. This video corresponds to the image sequence shown in Extended Data Fig. 3e.

Supplementary Video 9 Ultrasound-driven microbubble exhibiting a shape mode with an angular wavenumber *l* = 6. The field of view is 40 × 40 μm^2^ and the recording speed is 10 million frames per second. This video corresponds to the image sequence shown in Extended Data Fig. 3f.

Supplementary Video 10 Ultrasound-driven microbubble displaying jets induced by a shape mode with an angular wavenumber *l* = 1. The field of view is 40 × 40 μm^2^ and the recording speed is 10 million frames per second. This video corresponds to the image sequence shown in Extended Data Fig. 3h.

Supplementary Video 11 Ultrasound-driven microbubble displaying jets induced by a shape mode with an angular wavenumber *l* = 2. The field of view is 40 × 40 μm^2^ and the recording speed is 10 million frames per second. This video corresponds to the image sequence shown in Extended Data Fig. 3i.

Supplementary Video 12 Ultrasound-driven microbubble displaying jets induced by a shape mode with an angular wavenumber *l* = 3. The field of view is 40 × 40 μm^2^ and the recording speed is 10 million frames per second. This video corresponds to the image sequence shown in Extended Data Fig. 3j.

Supplementary Video 13 Ultrasound-driven microbubble displaying jets induced by a shape mode with an angular wavenumber *l* = 4. The field of view is 40 × 40 μm^2^ and the recording speed is 10 million frames per second. This video corresponds to the image sequence shown in Extended Data Fig. 3k.

Supplementary Video 14 Response of a 2.3-μm-radius microbubble in contact with a PEG substrate to a very intense ultrasound pulse (*f* = 1 MHz, *p*_a_ = 2.7 kPa, 20 cycles), captured from a side-view perspective. The bubble generates a single transient inertial jet followed by bubble fragmentation. The field of view is 40 × 40 μm^2^ and the recording speed is 10 million frames per second. This video corresponds to the image sequence shown in Extended Data Fig. 4.

Supplementary Video 15 Response of a 2.9-μm-radius microbubble in contact with an endothelial cell to an ultrasound pulse (*f* = 1 MHz, *p*_a_ = 150 kPa, 20 cycles), captured from a side-view perspective. Cyclic jetting is driven by a shape mode with wavenumber *l* = 1, as the bubble displays an alternate body motion. The field of view is 40 × 40 μm^2^ and the recording speed is 10 million frames per second. This video corresponds to the image sequence shown in Extended Data Fig. 5a.

Supplementary Video 16 Response of a 3.8-μm-radius microbubble in contact with an endothelial cell to an ultrasound pulse (*f* = 1 MHz, *p*_a_ = 80 kPa, 20 cycles), captured from a side-view perspective. Cyclic jetting is driven by a shape mode with wavenumber *l* = 2, as the bubble alternately takes on a prolate and oblate shape. The field of view is 40 × 40 μm^2^ and the recording speed is 10 million frames per second. This video corresponds to the image sequence shown in Extended Data Fig. 5b.

## Source data

Source Data Fig. 2 Experimental source data.

Source Data Fig. 3 Experimental source data.

Source Data Fig. 4 Experimental source data.

Source Data Extended Data Fig. 7 Experimental source data.

## Data Availability

Source data are provided with this paper. These data are also available via Zenodo at 10.5281/zenodo.14262735 (ref. ^100^).

## Code availability

The codes supporting this study are available via GitHub at https://github.com/cttnmrc/jetting-enables-sonoporation.git.
