# Supplementary material for: Cyclic jetting enables microbubble-mediated drug delivery
Source: Nat Phys. 2025 Feb 21;21(4):590–8. doi: 10.1038/s41567-025-02785-0 (PMC11999868; doi:10.1038/s41567-025-02785-0)
Supplement: Supplementary file 1 — Supplementary Methods and Description of supplementary videos. [file 41567_2025_2785_MOESM1_ESM.pdf]

nature physics

Supplementary information https://doi.org/10.1038/s41567-025-02785-0

Cyclic jetting enables microbubble-mediated drug delivery

In the format provided by the authors and unedited

Contents

1 Supplementary Information for Methods **2**
Microbubble preparation 2
PEG substrate preparation 2
Image analysis 2
Theoretical modelling of bubble radial dynamics 3

2 Supplementary Videos description **6**
Supplementary Video 1 6
Supplementary Video 2 6
Supplementary Video 3 6
Supplementary Video 4 6
Supplementary Video 5 6
Supplementary Video 6 7
Supplementary Video 7 7
Supplementary Video 8 7
Supplementary Video 9 7
Supplementary Video 10 7
Supplementary Video 11 7
Supplementary Video 12 7
Supplementary Video 13 8
Supplementary Video 14 8
Supplementary Video 15 8
Supplementary Video 16 8

1 Supplementary Information for Methods

Microbubble preparation

The lipids are first dissolved in chloroform, which is then let to evaporate under vacuum at 35 °C overnight, resulting in the formation of a dry lipid film. The film is rehydrated with PBS 1× (Boston BioProducts) to yield a total lipid concentration of $2\,\mathrm{mg\,ml^{-1}}$ and mixed using a probe sonicator (SFX550, Branson; 20 kHz, 550 W) at low power (30%). Microbubbles are formed by probe-sonicating the surface of the lipid solution at full power for ten seconds while simultaneously flowing C_4F_{10} gas over it. The microbubble suspension is then cooled down to room temperature and washed using centrifugation. Finally, differential centrifugation is employed to isolate microbubbles within a targeted size range (1 − 4 µm-radius) [5]. This size selection has been validated using a particle sizer (Multisizer 4e, Beckman Coulter), which is also used to measure the bubble concentration.

PEG substrate preparation

4-arm poly(ethylene glycol)-norbornene (PEG-NB) polymer precursor solution is mixed with matrix metalloproteinase (MMP)-cleavable peptide linker (KCGPQGI-WGQCK, Genscript) and arginylglycylaspartic acid cell adhesion peptide (CRGDS, Genscript) for a final hydrogel solution of 3.25 wt% PEG-NB, 2.35 mmol KCGPQGI-WGQCK, and 1.8 mmol CRGDS. To adjust for pH, a small volume of NaOH is added. This solution is then mixed with PBS and lithiumphenyl-2,4,6-tri-methylbenzoyl-phosphinate (LAP) photoinitiator to reach a final volume of 100 µL. Crosslinking is achieved through photoinitiated thiolene chemistry between the thiol groups of the MMP-cleavable linkers and the norbornene groups of PEG–NB. This is carried out under blue light exposure (λ = 405 nm, I = $14.5\,\mathrm{mW\,cm^{-2}}$, t = 90 s). Gel samples for AFM measurements are let to swell for twenty four hours in Dulbecco's modified Eagle's medium (DMEM) (Gibco, 1230-032) before testing. Capillary-hydrogel samples for experiments are stored in PBS until use.

Image analysis

In order to extract the time evolution of the microbubble radius and position from the bright-field recordings, individual frames are first filtered with a median filter to remove image noise and then binarised using locally adaptive thresholding. Subsequently, a flood-fill operation on holes in the binarised image is performed and all small noisy connected components are removed. The time-varying contour of the binarised bubble, denoted as $R(z,t)$ with z representing the axis normal to the substrate, is isolated. Since the bubble may undergo a non-spherical collapse—albeit predominantly axisymmetric—an equivalent bubble radius is derived by integrating the cross-sectional area, as follows:

$$R_{\mathrm{eq}}(t) = \left(\frac{3}{4} \int_{z^-}^{z^+} R(z,t)^2 dz \right)^{1/3}, \tag{1}$$

where z^- and z^+ represent the limiting values within which the bubble contour $R(z,t)$ is defined. Finally, the bubble position is extracted by computing the centroid of binarised bubble image.

Theoretical modelling of bubble radial dynamics

The dynamics of the liquid around the bubble is modelled using the Rayleigh–Plesset equation for mildly compressible Newtonian media [1], which reads:

$$\rho_\mathrm{l}\left(R\ddot{R}+\frac{3}{2}\dot{R}^2\right)=\left(1+\frac{R}{c_\mathrm{l}}\frac{d}{dt}\right)p_\mathrm{g}+\Sigma\left(R,\dot{R}\right)-p_\infty-p_\mathrm{d}\left(t\right)-4\mu_\mathrm{l}\frac{\dot{R}}{R}, \tag{2}$$

where over-dots denote time differentiation, $\rho_\mathrm{l}=997.8\,\mathrm{kg\,m^{-3}}$ is the liquid density, $c_\mathrm{l}=1481\,\mathrm{m\,s^{-1}}$ is the speed of sound in the medium, p_g is the gas pressure inside the bubble, $\Sigma(R,\dot{R})$ is the pressure term that accounts for the generalised interfacial stresses, $p_\infty=102.2\,\mathrm{kPa}$ is the undisturbed ambient pressure, $p_\mathrm{d}(t)$ is the ultrasound driving pressure and $\mu_\mathrm{l}=9.54\times10^{-4}\,\mathrm{Pa\,s}$ is the dynamic viscosity of the medium.

The ultrasound pressure $p_\mathrm{d}(t)$ can be expressed as the product of the signal amplitude p_a and the normalised time signal $\phi(t)$:

$$p_\mathrm{d}(t)=p_\mathrm{a}\phi(t). \tag{3}$$

$\phi(t)$ is measured experimentally using a hydrophone positioned at the ultrasound focal point without the presence of the test chamber, while p_a serves as the sole fitting parameter in our theoretical model. This indirect method of measuring the ultrasound pressure amplitude allows to account for the variable acoustic absorption caused by neighboring bubbles in the test chamber, as well as the acoustic reflections at the bubble's location. Direct measurement using a hydrophone would be impractical due to the minute scale of the problem. Regarding acoustic reflections, the rigid substrate used to cultivate the cells reflects part of the incoming acoustic wave, which then interferes with the incident wave, potentially with a phase shift. However, since the acoustic wavelength is over 100 times larger than the distance between the bubble and the substrate, this distance is negligible relative to the wavelength. As a result, the reflected wave remains in phase with the incident wave at the bubble's location. Thus, from the bubble's perspective, the rigid substrate merely amplifies the pressure of the incident wave, potentially doubling it if the substrate is a perfect reflector. Therefore, fitting the model based solely on the pressure amplitude p_a is sufficient to account for any acoustic reflections at the bubble's location.

The phospholipid coating reduces the gas-liquid surface tension, which decreases the large Laplace pressure at these bubble sizes, thereby halting gas efflux and prolonging the bubble's lifespan. Additionally, it imparts rheological properties to the interface [3]. The interfacial pressure term $\Sigma(R,\dot{R})$ related to the phospholipid coating is described with the Marmottant model [7], which is articulated as follows:

$$\Sigma\big(R,\dot{R}\big)=-2\frac{\sigma(R)}{R}-4\kappa_\mathrm{s}\frac{\dot{R}}{R^2},$$

$$\text{with} \quad \sigma(R) = \begin{cases} 0, & \text{for } R \leq R_{\text{buckling}}, \\ \sigma_0 + E_{\text{s}}\,(J-1), & \text{for } R_{\text{buckling}} < R \leq R_{\text{rupture}}, \\ \sigma_{\text{water}}, & \text{for } R > R_{\text{rupture}}, \end{cases} \tag{4}$$

where $\sigma(R)$ is the interfacial surface tension and κ_{s} is the interfacial dilatational viscosity. $\sigma(R)$ is a piecewise-defined function that accounts for: (i) the buckling of the coating, which occurs when the bubble is compressed to a radius smaller than R_{buckling}, resulting in a tension-free interface; (ii) the rupture of the coating, which occurs as the bubble expands beyond R_{rupture}, leading to the complete exposure of the gas core to the surrounding fluid, thus establishing a surface tension equivalent to that of a clean gas-water interface ($\sigma_{\text{water}} = 72.8\,\text{mN}\,\text{m}^{-1}$); and (iii) the elastic regime characterised by an interfacial dilatational modulus E_{s} that lies in between. σ_0 is the interfacial surface tension at equilibrium and $J = R^2/R_0^2$ is the relative area deformation. The values for the shell rheological parameters are specified based on our prior rheological investigation [2] and read $\kappa_{\text{s}} = 5 \times 10^{-9}\,\text{kg}\,\text{s}^{-1}$, $E_s = 0.2\,\text{N}\,\text{m}^{-1}$ and $\sigma_0 = 0\,\text{N}\,\text{m}^{-1}$. A tensionless bubble at equilibrium is consistent with its observed long-term stability against dissolution in a saturated medium [4].

For microbubbles driven by ultrasound at megahertz frequencies, the validity of the commonly-used polytropic process approximation for the bubble gas pressure p_{g} is typically violated because the Péclet number is close to one [8]. Therefore, we employ Zhou's model [9] to address the thermal interaction problem which governs the bubble gas pressure p_{g}. The method is based on the well-accepted assumption that the gas pressure is uniform within the bubble [8]. This allows, for a perfect gas, to exactly express the gas radial velocity as:

$$u_{\text{g}}(r) = \frac{1}{\gamma p_{\text{g}}} \left[(\gamma - 1)\, K_{\text{g}} \frac{\partial T_{\text{g}}}{\partial r} - \frac{1}{3} r \dot{p}_g \right], \tag{5}$$

and from this result recover an exact relation for the gas pressure:

$$\dot{p}_{\text{g}} = \frac{3}{R} \left[(\gamma - 1)\, K_{\text{g}} \left.\frac{\partial T_{\text{g}}}{\partial r}\right|_R - \gamma p_{\text{g}} \dot{R} \right], \tag{6}$$

where r is the radial coordinate, γ is the gas specific heat ratio, K_{g} is the gas thermal conductivity and T_{g} is the gas temperature. The temperature profile $T_{\text{g}}(r)$ inside the bubble is divided into three regions: (1) an internal layer characterised by uniform temperature, (2) a buffer layer, and (3) an external layer characterised by a linear temperature distribution. The change of bubble surface temperature is significantly smaller compared to that of the gas and can, therefore, be neglected, i.e. $T_{\text{g}}|_R \approx T_{\text{l}}$ [8]. The volume-averaged temperature T_{g_i} of each region i can be computed through the equation of state for an ideal gas:

$$T_{\text{g}_i} = \frac{p_{\text{g}}}{\rho_{\text{g}_i} \mathcal{R}}, \quad \text{for } i = 1, 2, 3, \tag{7}$$

where ρ_{g_i} is the volume-averaged gas density of the region i and $\mathcal{R}$ is the specific gas constant. ρ_{g_i} can be computed using the continuity equation for each region:

$$\dot{m}_{g,1} = -f_1, \quad \dot{m}_{g,2} = f_1 - f_2, \quad \dot{m}_{g,3} = f_2, \tag{8}$$

where m_{g_i} is the gas mass in region i and f_j is the mass flux across the region interface j, which has the form:

$$f_j = \rho^{\mathrm{uw}}_{g,j} u^{\mathrm{rel}}_{g,j} S_j, \quad \text{for } j = 1, 2, \tag{9}$$

where $\rho^{\mathrm{uw}}_{g,j}$ is the density of the neighbouring cell on the upwind side of interface j, $u^{\mathrm{rel}}_{g,j}$ is the convective velocity, which is the difference between the real velocity of the gas, as defined in Eq. (5), and the velocity of the cell interface j, and S_j is the surface area of the interface j. Due to the long residence time (approximately ten minutes) in an air-saturated environment, the microbubble gas core is replaced by air [6]. Therefore, the values for the gas parameters are $\gamma = 1.4$, $K_g = 0.026\,\mathrm{W\,m^{-1}\,K^{-1}}$ and $\mathcal{R} = 287\,\mathrm{J\,kg^{-1}\,K^{-1}}$.

References

[1] M. P. Brenner, S. Hilgenfeldt, and D. Lohse. Single-bubble sonoluminescence. *Reviews of Modern Physics*, 74(2):425–484, 2002.

[2] M. Cattaneo and O. Supponen. Shell viscosity estimation of lipid-coated microbubbles. *Soft Matter*, 19(31):5925–5941, 2023.

[3] D. A. Edwards, H. Brenner, and D. T. Wasan. *Interfacial Transport Processes and Rheology.* Elsevier, 1991.

[4] K. Ferrara, R. Pollard, and M. Borden. Ultrasound microbubble contrast agents: Fundamentals and application to gene and drug delivery. *Annual Review of Biomedical Engineering*, 9:415–447, 2007.

[5] J. A. Feshitan, C. C. Chen, J. J. Kwan, and M. A. Borden. Microbubble size isolation by differential centrifugation. *Journal of Colloid and Interface Science*, 329(2):316–324, 2009.

[6] J. J. Kwan and M. A. Borden. Lipid monolayer dilatational mechanics during microbubble gas exchange. *Soft Matter*, 8(17):4756–4766, 2012.

[7] P. Marmottant, S. Van Der Meer, M. Emmer, M. Versluis, N. De Jong, S. Hilgenfeldt, and D. Lohse. A model for large amplitude oscillations of coated bubbles accounting for buckling and rupture. *Journal of the Acoustical Society of America*, 118(6):3499–3505, 2005.

[8] A. Prosperetti, L. A. Crum, and K. W. Commander. Nonlinear bubble dynamics. *Journal of the Acoustical Society of America*, 83(2):502–514, 1988.

[9] G. Zhou. Modeling the thermal behavior of an acoustically driven gas bubble. *Journal of the Acoustical Society of America*, 149(2):923–933, 2021.

2 Supplementary Videos description

Supplementary Video 1

Response of a 3 µm-radius microbubble in contact with an endothelial cell to an ultrasound pulse ($f = 1$ MHz, $p_a = 60$ kPa, 20 cycles), captured from a side-view perspective. The amplitude of the applied ultrasound pressure is insufficient to induce the formation of cyclic piercing microjets from the microbubble. The field of view is 40×40 µm and the recording speed is 10 million frames per second. This video corresponds to the image sequence shown in Fig. 1c.

Supplementary Video 2

Response of the same 3 µm-radius microbubble in contact with an endothelial cell to a more intense ultrasound pulse ($f = 1$ MHz, $p_a = 160$ kPa, 20 cycles), captured from a side-view perspective. The higher amplitude of the applied ultrasound pressure leads to the formation of cyclic piercing microjets directed towards the cell, resulting in cell membrane poration and drug uptake. The bubble motion also results in the formation of a transendothelial tunnel. The field of view is 40×40 µm and the recording speed is 10 million frames per second. This video corresponds to the image sequence shown in Fig. 1d.

Supplementary Video 3

Response of a 3 µm-radius microbubble in contact with an endothelial cell to an ultrasound pulse ($f = 1$ MHz, $p_a = 175$ kPa, 20 cycles), captured from a side-view perspective. In this instance, the bubble generates cyclic piercing microjets that facilitate drug uptake, but the motion of the bubble does not result in the formation of a transendothelial tunnel. The field of view is 40×40 µm and the recording speed is 10 million frames per second. This video corresponds to the image sequence shown in Extended Data Fig. 2.

Supplementary Video 4

Ultrasound-driven microbubble exhibiting a shape mode with an angular wavenumber $l = 1$. The field of view is 40×40 µm and the recording speed is 10 million frames per second. This video corresponds to the image sequence shown in Extended Data Fig. 3a.

Supplementary Video 5

Ultrasound-driven microbubble exhibiting a shape mode with an angular wavenumber $l = 2$. The field of view is 40×40 µm and the recording speed is 10 million frames

per second. This video corresponds to the image sequence shown in Extended Data Fig. 3b.

Supplementary Video 6

Ultrasound-driven microbubble exhibiting a shape mode with an angular wavenumber $l = 3$. The field of view is $40 \times 40\,\mu\mathrm{m}$ and the recording speed is 10 million frames per second. This video corresponds to the image sequence shown in Extended Data Fig. 3c.

Supplementary Video 7

Ultrasound-driven microbubble exhibiting a shape mode with an angular wavenumber $l = 4$. The field of view is $40 \times 40\,\mu\mathrm{m}$ and the recording speed is 10 million frames per second. This video corresponds to the image sequence shown in Extended Data Fig. 3d.

Supplementary Video 8

Ultrasound-driven microbubble exhibiting a shape mode with an angular wavenumber $l = 5$. The field of view is $40 \times 40\,\mu\mathrm{m}$ and the recording speed is 10 million frames per second. This video corresponds to the image sequence shown in Extended Data Fig. 3e.

Supplementary Video 9

Ultrasound-driven microbubble exhibiting a shape mode with an angular wavenumber $l = 6$. The field of view is $40 \times 40\,\mu\mathrm{m}$ and the recording speed is 10 million frames per second. This video corresponds to the image sequence shown in Extended Data Fig. 3f.

Supplementary Video 10

Ultrasound-driven microbubble displaying jets induced by a shape mode with an angular wavenumber $l = 1$. The field of view is $40 \times 40\,\mu\mathrm{m}$ and the recording speed is 10 million frames per second. This video corresponds to the image sequence shown in Extended Data Fig. 3h.

Supplementary Video 11

Ultrasound-driven microbubble displaying jets induced by a shape mode with an angular wavenumber $l = 2$. The field of view is $40 \times 40\,\mu\mathrm{m}$ and the recording speed is 10 million frames per second. This video corresponds to the image sequence shown in Extended Data Fig. 3i.

Supplementary Video 12

Ultrasound-driven microbubble displaying jets induced by a shape mode with an angular wavenumber $l = 3$. The field of view is $40 \times 40\,\mu\mathrm{m}$ and the recording speed is 10

million frames per second. This video corresponds to the image sequence shown in Extended Data Fig. 3j.

Supplementary Video 13

Ultrasound-driven microbubble displaying jets induced by a shape mode with an angular wavenumber $l = 4$. The field of view is $40 \times 40\,\mu m$ and the recording speed is 10 million frames per second. This video corresponds to the image sequence shown in Extended Data Fig. 3k.

Supplementary Video 14

Response of a $2.3\,\mu m$-radius microbubble in contact with a PEG substrate to a very intense ultrasound pulse ($f = 1\,MHz$, $p_a = 2.7\,MPa$, 20 cycles), captured from a side-view perspective. The bubble generates a single transient inertial jet followed by bubble fragmentation. The field of view is $40 \times 40\,\mu m$ and the recording speed is 10 million frames per second. This video corresponds to the image sequence shown in Extended Data Fig. 4.

Supplementary Video 15

Response of a $2.9\,\mu m$-radius microbubble in contact with an endothelial cell to an ultrasound pulse ($f = 1\,MHz$, $p_a = 150\,kPa$, 20 cycles), captured from a side-view perspective. Cyclic jetting is driven by a shape mode with wavenumber $l = 1$, as the bubble displays an alternate body motion. The field of view is $40 \times 40\,\mu m$ and the recording speed is 10 million frames per second. This video corresponds to the image sequence shown in Extended Data Fig. 5a.

Supplementary Video 16

Response of a $3.8\,\mu m$-radius microbubble in contact with an endothelial cell to an ultrasound pulse ($f = 1\,MHz$, $p_a = 80\,kPa$, 20 cycles), captured from a side-view perspective. Cyclic jetting is driven by a shape mode with wavenumber $l = 2$, as the bubble alternately takes on a prolate and oblate shape. The field of view is $40 \times 40\,\mu m$ and the recording speed is 10 million frames per second. This video corresponds to the image sequence shown in Extended Data Fig. 5b.
