# Supplementary material for: Cyclic jetting enables microbubble-mediated drug delivery
Source: Nat Phys. 2025 Feb 21;21(4):590–8. doi: 10.1038/s41567-025-02785-0 (PMC11999868; doi:10.1038/s41567-025-02785-0)
Supplement: Supplementary file 2 — Reporting Summary [file 41567_2025_2785_MOESM2_ESM.pdf]

nature portfolio

Corresponding author(s): Marco Cattaneo

Last updated by author(s): Dec 3, 2024

Reporting Summary

Nature Portfolio wishes to improve the reproducibility of the work that we publish. This form provides structure for consistency and transparency in reporting. For further information on Nature Portfolio policies, see our Editorial Policies and the Editorial Policy Checklist.

Statistics

For all statistical analyses, confirm that the following items are present in the figure legend, table legend, main text, or Methods section.

n/a	Confirmed	
☐	☒	The exact sample size (n) for each experimental group/condition, given as a discrete number and unit of measurement
☐	☒	A statement on whether measurements were taken from distinct samples or whether the same sample was measured repeatedly
☒	☐	The statistical test(s) used AND whether they are one- or two-sided<br>*Only common tests should be described solely by name; describe more complex techniques in the Methods section.*
☒	☐	A description of all covariates tested
☒	☐	A description of any assumptions or corrections, such as tests of normality and adjustment for multiple comparisons
☐	☒	A full description of the statistical parameters including central tendency (e.g. means) or other basic estimates (e.g. regression coefficient) AND variation (e.g. standard deviation) or associated estimates of uncertainty (e.g. confidence intervals)
☒	☐	For null hypothesis testing, the test statistic (e.g. F, t, r) with confidence intervals, effect sizes, degrees of freedom and P value noted<br>*Give P values as exact values whenever suitable.*
☒	☐	For Bayesian analysis, information on the choice of priors and Markov chain Monte Carlo settings
☒	☐	For hierarchical and complex designs, identification of the appropriate level for tests and full reporting of outcomes
☒	☐	Estimates of effect sizes (e.g. Cohen's d, Pearson's r), indicating how they were calculated

Our web collection on statistics for biologists contains articles on many of the points above.

Software and code

Policy information about availability of computer code

Data collection	Video recordings were captured using a Shimadzu HPV-X2 camera operated through its dedicated control software.<br>Hydrophone measurements were conducted using a Teledyne LeCroy WaveRunner 9404 oscilloscope via its integrated software.<br>The compression elastic modulus of PEG hydrogel substrates was measured using a Nanosurf Flex-Bio atomic force microscope via its integrated control software.
Data analysis	MATLAB_r2023a. The source codes are are available via GitHub at https://github.com/cttnmrc/jetting-enables-sonoporation.git

For manuscripts utilizing custom algorithms or software that are central to the research but not yet described in published literature, software must be made available to editors and reviewers. We strongly encourage code deposition in a community repository (e.g. GitHub). See the Nature Portfolio guidelines for submitting code & software for further information.

Data

Policy information about availability of data

All manuscripts must include a data availability statement. This statement should provide the following information, where applicable:

- Accession codes, unique identifiers, or web links for publicly available datasets
- A description of any restrictions on data availability
- For clinical datasets or third party data, please ensure that the statement adheres to our policy

Source data supporting this study are provided with this paper and via Zenodo at https://doi.org/10.5281/zenodo.14262735.

Research involving human participants, their data, or biological material

Policy information about studies with human participants or human data. See also policy information about sex, gender (identity/presentation), and sexual orientation and race, ethnicity and racism.

Reporting on sex and gender	n/a
Reporting on race, ethnicity, or other socially relevant groupings	n/a
Population characteristics	n/a
Recruitment	n/a
Ethics oversight	n/a

Note that full information on the approval of the study protocol must also be provided in the manuscript.

Field-specific reporting

Please select the one below that is the best fit for your research. If you are not sure, read the appropriate sections before making your selection.

☒ Life sciences ☐ Behavioural & social sciences ☐ Ecological, evolutionary & environmental sciences

For a reference copy of the document with all sections, see nature.com/documents/nr-reporting-summary-flat.pdf

Life sciences study design

All studies must disclose on these points even when the disclosure is negative.

Sample size	The number of tests conducted (n=37) constitutes a convenience sample, determined by the feasibility of completing them within the study's allotted timeframe and budget.<br>In 19 tests where bubble jets were observed, sonoporation of the cell membrane also occurred, achieving a 100% success rate. This provides compelling evidence of a strong relationship between the presence of bubble jets and sonoporation. While technically larger samples could further narrow uncertainty, the absence of failures suggests diminishing returns from additional tests.
Data exclusions	No data was excluded.
Replication	Most tests acted as biological replicates since they were conducted on distinct cell monolayer samples. Sequential tests using the same bubble on the same cell were conducted whenever conditions permitted, aiming to determine the threshold ultrasound parameters necessary for effective sonoporation. The specific test groups included: [test 1, 2, 3], [test 4, 5], [test 11, 12, 17], [test 13, 18, 21], [test 14, 16], [test 15, 20], [test 19, 23, 29], [test 22, 33], [test 25, 28], [test 26, 35, 36, 37], [test 27, 32], and [test 30, 31, 34]. Please refer to "Cattaneo_Fig_4.xlsx" source file for more information.
Randomization	The pairing of a cell and a bubble is inherently random, as it is impossible to control which bubble, through flotation, will come into contact with which cell. The selection of a bubble-cell combination is determined solely by factors such as image quality and lighting conditions.
Blinding	The experimental recording of microbubble dynamics were analysed to evaluate the ultrasound pressure, the radial expansion and the jetting occurrence using coded identifiers, without linking them to the sonoporation outcome.

Reporting for specific materials, systems and methods

We require information from authors about some types of materials, experimental systems and methods used in many studies. Here, indicate whether each material, system or method listed is relevant to your study. If you are not sure if a list item applies to your research, read the appropriate section before selecting a response.

Materials & experimental systems

n/a	Involved in the study
☐	☒ Antibodies
☐	☒ Eukaryotic cell lines
☒	☐ Palaeontology and archaeology
☒	☐ Animals and other organisms
☒	☐ Clinical data
☒	☐ Dual use research of concern
☒	☐ Plants

Methods

n/a	Involved in the study
☒	☐ ChIP-seq
☒	☐ Flow cytometry
☒	☐ MRI-based neuroimaging

Antibodies

Antibodies used	Primary antibody:<br>Mouse anti-CD31 primary antibody (BD Biosciences, Cat. Nr. 555444, monoclonal, clone WM59, used at 1:200 dilution).<br><br>Secondary antibody:<br>AlexaFluor 488 goat-anti-mouse secondary antibody (abcam, Cat. Nr. ab150113, polyclonal, used at 1:200 dilution)
Validation	Primary antibody:<br>Validated for flow cytometry by manufacturers (BD Biosciences). It has been used for ICC in several publications.<br>Authors confirmed absence of signal if primary antibody was omitted (negative staining control).<br><br>Secondary antibody:<br>Validated for ICC and other applications by manufacturers (abcam).

Eukaryotic cell lines

Policy information about cell lines and Sex and Gender in Research

Cell line source(s)	Cat. Nr. C-12200, PromoCell GmbH, Lot. 4032501.1, female
Authentication	The HUVECs used were not formally authenticated by the authors but retained the expected endothelial morphology (cobblestone-like monolayers) throughout culture and stained positive for endothelial marker CD31.
Mycoplasma contamination	Negative for mycoplasma as evidenced by DAPI staining. PCR-test for mycoplasma was not done for these cells.
Commonly misidentified lines<br>(See ICLAC register)	No commonly misidentified cell lines were used.

Plants

Seed stocks	n/a
Novel plant genotypes	n/a
Authentication	n/a
